# Spatiotemporal Distribution of *Ulva* Micropropagules in Surface Sediments: Insights from Field Sampling Data in the Southern Yellow Sea (2020–2022)

**DOI:** 10.3390/biology15151220

**Published:** 2026-07-23

**Authors:** Yutao Qin, Lihua Xia, Huanhong Ji, Xiaobo Wang, Jiaxing Cao, Yuhan Zhang, Jing Xia, Jinlin Liu

**Affiliations:** 1 Key Laboratory of Marine Ecological Monitoring and Restoration Technologies, East China Sea Ecological Center, Ministry of Natural Resources, Shanghai 201206, China; qinyutaox@163.com (Y.Q.); oucwangxb@163.com (X.W.); jiaxingcao910@gmail.com (J.C.); zhangyh266@foxmail.com (Y.Z.); 2 School of Oceanography, Shanghai Jiao Tong University, Shanghai 200030, China; xiajingsherry@sjtu.edu.cn; 3 Project Management Office of China National Scientific Seafloor Observatory, Tongji University, Shanghai 200092, China

**Keywords:** the Yellow Sea, sediments, in situ investigation, green tides, microscopic propagules, aquaculture industry

## Abstract

Green tide disasters have erupted consecutively in the Southern Yellow Sea, yet their driving mechanisms remain debated. The “raft-origin-hypothesis” identifies the source as epiphytic *Ulva* macroalgae attached to laver aquaculture rafts in the Subei Shoal, whereas the “micropropagule-hypothesis” speculates that *Ulva* micropropagules settling into open sea sediments constitute a cross-year seed reserve. The latter has hitherto relied primarily on laboratory culture simulations, with field-scale assessment lacking. This study established 46 surface sediment survey stations from 2020 to 2022, covering periods before, during, and after green tide outbreaks. Surface sediments were obtained using a box corer, and *Ulva* micropropagules were quantitatively analyzed through laboratory cultivation and morphological identification. Results showed that micropropagule abundance was generally low and exhibited spatial heterogeneity; high-value areas persistently existed in the Subei Shoal aquaculture zone, extended into the open sea during outbreaks, and generally disappeared in the open sea during and after the decline period. Monthly variations indicated that micropropagules released and settling into sediments (e.g., in the open sea) during large-scale outbreaks could not survive until the following year’s floating period; the initial biomass of subsequent eruptions did not originate from the revival of the seafloor “seed source” in the open sea, which remains speculative.

## Simple Summary

In recent years, the academic community has questioned the “raft-origin-hypothesis”, and a small number of scholars have proposed that micropropagules of the genus *Ulva*, which may be widely present and persist in surface sediments of the Southern Yellow Sea, could be the hidden driver behind the annual recurrence of green tides. This study conducted a three-year continuous tracking survey of stations in the Southern Yellow Sea; by collecting surface sediments from the seafloor and cultivating them under laboratory conditions, we systematically revealed the spatiotemporal distribution patterns of these *Ulva* micropropagules. The survey found that the dense areas of *Ulva* micropropagules were consistently located near the laver aquaculture zones in the Subei Shoal, while they were almost undetectable in the open sea after the green tide subsided. This suggests that the open sea may not be a significant driver of green tide recurrence, and the revival of the “seed source” on the seafloor of the open sea, as speculated by some scholars, is likely not the primary source of the initial biomass for green tides erupting in the following year. These findings provide a new scientific perspective for the precise prevention and control of green tide disasters.

## 1. Introduction

The green tide disaster in the Southern Yellow Sea has been recurring for two decades, posing a significant threat to regional marine ecology and economy. During algal bloom outbreaks, green tide algae (at least five *Ulva* species, with *Ulva prolifera* O.F. Müller, 1778 being the most typical) proliferate massively, shading the sea surface, altering water optical properties, inhibiting phytoplankton photosynthesis, and disrupting the relatively stable primary productivity structure and biodiversity of the original sea area [1,2,3,4]; during their accumulation or decomposition stages, they cause localized water hypoxia and the release of toxic metabolic byproducts such as sulfides, subsequently exacerbating the degradation risk of marine ecosystems (e.g., benthic organisms) [5,6,7]. Meanwhile, green tide disasters cause direct economic losses to coastal tourism landscape quality and aquaculture, and have become one of the important marine ecological disasters constraining regional marine sustainable development [8].

Regarding the core cause of green tide outbreaks in the Southern Yellow Sea, the current mainstream academic view tends toward the “raft-origin-hypothesis” [9]: epiphytic *Ulva* green algae attached to laver aquaculture rafts in the Subei Shoal are considered the key source triggering green tides; the attached *Ulva prolifera* on them accounts for a certain proportion of biomass [10,11], resulting in a considerable total biomass of attached *Ulva prolifera* on the rafts. Under the dual background of marine eutrophication and floating raft aquaculture, epiphytic green algae on rafts detach due to environmental factors or human disturbance, proliferate massively under suitable hydrometeorological conditions, and are continuously transported to Jiangsu and Shandong sea areas driven by wind and ocean currents, ultimately forming large-scale green tide disasters that severely impact the coastal ecological environment [12,13,14]. Some scholars have also pointed out that although the scale of epiphytic *Ulva* green algae colonizing intertidal wetlands and estuarine channels is relatively limited, their natural detachment process can provide a certain initial biomass supplement for green tide outbreaks, constituting a potential secondary source [15,16,17,18].

In addition, a minority of researchers believe that coastal mariculture ponds in Jiangsu Province (mainly for aquatic animal culture) make a key contribution, and have noted that the attached or floating *Ulva prolifera* in ponds share highly similar genotypic characteristics with the floating *Ulva prolifera* during green tide outbreaks in the Southern Yellow Sea [19]; moreover, farmers’ pond cleaning behavior during the culture cycle leads to partial artificial release of algal bodies, and during tidal rise and fall farmers complete partial water exchange in the culture water [20], with the cleaned *Ulva* green algae discharged into the sea through drainage channels. Consequently, some scholars believe that this portion of *Ulva* algal bodies is likely an important source causing green tide disasters in the Southern Yellow Sea. Controversy remains regarding the core source of green tides, but the “raft-origin-hypothesis” still dominates the academic community, with subsequent research mostly focusing on source tracing verification, deepening of outbreak mechanisms, and source prevention and control studies based on this hypothesis [21,22,23,24,25], and the source prevention and control, process salvage, and beach cleanup of green tides in the Southern Yellow Sea have also been advanced accordingly [21,26].

Although source prevention and control efforts have achieved certain results [21,26], because green tides continue to erupt, the “raft-origin-hypothesis” has gradually been questioned by some scholars in the past eight years. Some researchers have proposed another potential mechanism—the “micropropagule-hypothesis”: the annual recurrence of green tides may be closely related to *Ulva* micropropagules that have settled to the seafloor of the open sea over the years (referring to all biological units produced by *Ulva* algae that have the potential to develop into new algal bodies, including spores, gametes, and zygotes at the single-cell level, microscopic individuals at different developmental stages, and macroscopic-scale invisible tissue fragments of macroalgae that still have regenerative capacity under suitable conditions [27,28]). Some experts have proposed that *Ulva* micropropagules can survive in dark sediment environments for 3–5 years while maintaining germination capacity [28]; researchers believe that theoretically, when environmental conditions are suitable (such as spring water temperature rise and sufficient nutrients), micropropagules in the open sea sediments can be resuspended into the water column under hydrodynamic disturbance and germinate and proliferate (before the outbreak of green tide disasters in the Southern Yellow Sea, suspended *Ulva* macroalgae can indeed be collected in the water column [29]), thereby providing initial sources for a new round of green tide disasters [28,30]. However, this hypothesis is currently mainly based on laboratory culture evidence [28,31], and systematic field surveys and direct verification regarding the actual stock and spatiotemporal distribution characteristics of *Ulva* micropropagules in the Southern Yellow Sea are still lacking.

Therefore, this study conducted systematic surveys in the Southern Yellow Sea from 2020 to 2022 for three consecutive years, covering three key stages: before green tide outbreaks, during the outbreak period, and during (or after) the decline period. By combining sediment sample collection with laboratory cultivation, quantitative analysis of the spatiotemporal distribution characteristics and density dynamics of *Ulva* micropropagules in surface sediments was performed. The study area covered the Subei Shoal to key sea areas in northern Jiangsu, with a total of 46 survey stations established (including fixed monitoring stations and temporary monitoring stations), aiming to clarify: (1) the abundance level and spatial distribution pattern of *Ulva* micropropagules in Southern Yellow Sea sediments; (2) the monthly variation patterns of micropropagule density and their potential association with the green tide outbreak process. The results will provide a theoretical basis and data support for evaluating the scientific validity of the “micropropagule-hypothesis”, improving early warning mechanisms for green tide disasters, formulating targeted source prevention and control strategies, and advancing future research directions.

## 2. Materials and Methods

### 2.1. Regional Geographic Background, Sampling Station Layout, and Specific Sampling Times

The Southern Yellow Sea is located in eastern China, between the Shandong Peninsula, the Jiangsu coast, and the Korean Peninsula, belonging to the southern part of the Yellow Sea. It has relatively shallow water depth and relatively flat seafloor topography, belonging to a continental shelf shallow sea. Influenced by the East Asian monsoon, Changjiang (Yangtze River) runoff, and the Yellow Sea Warm Current, the area also experiences significant coastal currents and tidal activities. The seawater in this region exhibits a relatively high degree of eutrophication. Additionally, it serves as an important traditional fishing ground and an area of oil and gas resource distribution in China; it is also an ecologically sensitive sea area where typical marine ecological disasters such as red tides, green tides, and golden tides frequently occur [32,33,34,35,36].

**Figure 1:**
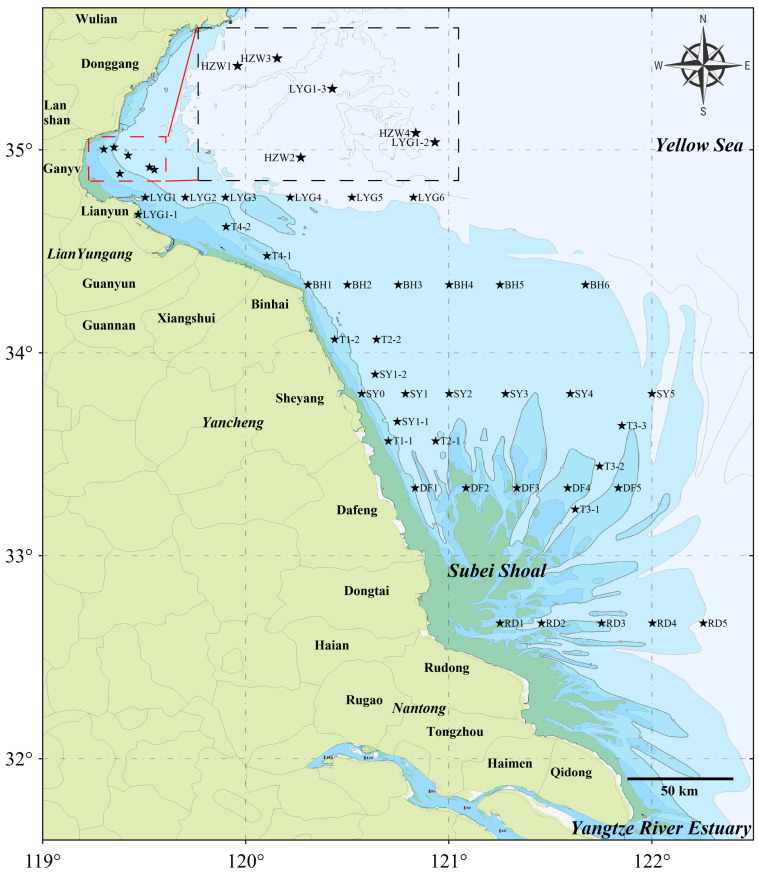
Spatial distribution of sampling stations in the Southern Yellow Sea.

This study established a total of 46 survey stations in the Southern Yellow Sea, including 23 fixed monitoring stations and 23 temporary monitoring stations; the specific spatial distribution is shown in Figure 1. All sampling work was completed on marine survey vessels chartered by the Ministry of Natural Resources; the vessels used were “Zheding Yuyun 11190”, “Binhai Yu 15045”, and “Baohai Jiaoce 6”. Concurrently, wind speed and wind direction data were continuously recorded by the XZC6-1 shipboard automatic weather station (National Ocean Technology Center, Tianjin, China) mounted on each vessel during the survey cruises. During sampling, shipboard echo sounders were used to obtain actual water depth data at each station to provide a reference for sediment sample collection. Subsequently, the DDC2-1 box corer (Qingdao Lanke Marine Instrument Equipment Co., Ltd., Qingdao, China) was deployed at a controlled descent rate (approximately 0.2 m/s) to minimize seabed disturbance upon contact. Upon penetration, the closure mechanisms sealed the sample chamber, thereby minimizing the loss of overlying water and fine surface sediments during recovery. Only samples with clear overlying water, undisturbed sediment surfaces, and complete closure of the sealing mechanisms were retained for further processing. After collection, a high-temperature sterilized stainless steel spatula was used to scrape the surface (0–2 cm) sediments, which were immediately placed into sterile sealed polyethylene bags.

The specific sampling times of this study covered April, May, and July 2020; March, May, and November 2021; and March, May, and December 2022. During this period, surface sediment samples were routinely collected at 23 fixed monitoring stations (stations RD1–RD5, SY1–SY3, DF1–DF5, BH1–BH5, LYG1–LYG5). To capture the dynamics of *Ulva* micropropagules across the green tide cycle (before the outbreak, during the outbreak period, and after decline, temporary monitoring stations were strategically added at different time points. In November 2021, 9 temporary monitoring stations were established (stations SY0, SY1-1, SY1-2, SY4, SY5, BH6, LYG1-1, LYG1-2, LYG1-3) to assess post-decline background levels in the main affected areas. In 2022, more temporary stations were added compared to previous years: in March 2022, 8 temporary monitoring stations (stations SY0, SY1-1, SY1-2, SY4, LYG1-1, LYG1-2, LYG1-3, LYG6) were added to track pre-outbreak overwintering micropropagules; in May 2022, 2 temporary monitoring stations (stations SY0, SY4) were established to monitor micropropagule dynamics during the outbreak; and in December 2022, 15 temporary monitoring stations (stations SY0, SY4, HZW1–HZW4, T1-1, T1-2, T2-1, T2-2, T3-1, T3-2, T3-3, T4-1, T4-2) were deployed for an expanded post-decline survey (Figure 1; Table 1).

### 2.2. Micropropagule Cultivation Procedure from Sediments

Surface sediment samples were stored at 4 °C in the dark and immediately transported back to the laboratory. First, they were observed under a dissecting microscope to determine whether obvious macroalgal fragments were present in the sediment; if fragments existed, they were promptly removed. Subsequently, 20 g of surface sediment sample (wet weight) was placed in a 3 L glass beaker (before the experiment, the 3 L glass beaker was pre-soaked in 10% HCl for 10 h, then thoroughly rinsed with distilled water), then 2 L of autoclaved and cooled artificial seawater with a salinity of 25 was added to the glass beaker, stirred thoroughly with a glass rod, and left to stand for 30 min. Afterward, the relatively clear supernatant turbid liquid from the upper part of the beaker was transferred to another sterilized 3 L glass beaker, artificial seawater was added to bring the volume to 2 L, and the larger stones and sand grains that had settled to the bottom of the original glass beaker were discarded. After stirring thoroughly and evenly again with a glass rod, 100 mL of turbid liquid was taken and transferred into an autoclaved 1 L glass beaker, then 400 mL of autoclaved and cooled artificial seawater was added (this procedure was repeated to prepare three parallel samples for each station) [37,38].

1 mL of saturated GeO_2_ solution was added to the cultivation system to inhibit microalgal growth. An additional 1 mL of von Stosch’s Enriched (VSE) nutrient solution (the prepared nutrient solution also needed to be autoclaved and cooled to room temperature before use) was added as a nutrient substance to promote algal growth [12,39]; the cultivation system was stirred evenly again with a glass rod, with continuous stirring for at least 60 s. Subsequently, the prepared surface sediment cultivation system was placed in an illumination incubator for static cultivation at a temperature of 18 °C, light intensity of 50–100 μmol m^−2^ s^−1^, and a photoperiod of 12 L:12 D [27]. During the early cultivation period (days 0–7), the light intensity was set to 50 μmol m^−2^ s^−1^; after algal germination (days 7–14), the light intensity was set to 80 μmol m^−2^ s^−1^; during the rapid algal growth period (days 14–21), the light intensity was set to 100 μmol m^−2^ s^−1^. To support propagule growth, 2 mL of VSE medium was supplemented every 4 days. After approximately 21 days, macroscopic algae visible to the naked eye, developed from germinated micropropagules, could be observed on the inner walls and bottom of the beaker (if a large number of microalgae appeared in the cultivation system during algal growth, two effective methods could be adopted to inhibit microalgal growth and reproduction: first, continue to add 1 mL of saturated GeO_2_ solution to the cultivation system; second, remove the macroalgae, use a brush to remove epiphytic microalgae from their surface, and then transfer the macroalgae to a new 1 L glass beaker cultivation system containing 500 mL of artificial seawater and VSE nutrient solution). Subsequently, the macroalgae were removed from the cultivation system for further morphological observation and species identification.

### 2.3. Morphological Identification and Quantification

When the micropropagules cultivated from surface sediments grew to a length ≥ 10 cm (the cultivation cycle was generally approximately 21–30 days), preliminary identification of mature macroalgal individuals was conducted based on morphological characteristics to confirm whether the cultivated micropropagules belonged to the genus *Ulva*. The morphological indicators referenced for identification included: thallus shape, whether the base had a discoid prostrate structure, branching pattern, rhizoid characteristics, cell layer number, nucleus number, chloroplast quantity and morphology, and pyrenoid number. The identification standards were mainly based on Ding & Luan [40]. To ensure the scientific reliability of the taxonomic results, senior domestic phycologists were subsequently invited to conduct secondary confirmation of the specimens based on classical taxonomic systems and taxonomic experience, further verifying whether they belonged to the genus *Ulva*. On this basis, the total number of *Ulva* individuals from each sampling station was counted, and the abundance of *Ulva* micropropagules in surface sediment samples was calculated, with results expressed as individuals per gram of sediment (inds g^−1^).

### 2.4. Data Analysis and Figure Plotting

To investigate the spatial distribution pattern of micropropagules in surface sediments, professional marine data processing and visualization software was used for systematic analysis. The specific procedure was as follows: first, Origin 2021 software was used to conduct descriptive statistics on the micropropagule abundance data from each sampling station, calculating the mean abundance and standard deviation (SD) for all stations, expressed in the form of “mean ± standard deviation”. Subsequently, the mean abundance of each station was used as the basic data for spatial interpolation, imported into Surfer 9 software, and the Kriging interpolation algorithm was selected for spatial analysis and calculation to identify overall distribution trends and regional heterogeneity. Based on the interpolation results, contour maps of micropropagule density were plotted to visualize the spatial distribution characteristics.

For the supplementary wind field visualization, wind speed and wind direction data recorded at each sampling station were compiled and processed using Python 3.9 with the Matplotlib library (version 3.7.1). The wind direction data (recorded in degrees, where 0° indicates wind blowing from the north) were classified into 16 directional sectors (N, NNE, NE, ENE, E, ESE, SE, SSE, S, SSW, SW, WSW, W, WNW, NW, NNW) at 22.5° intervals. Wind speed was categorized into five classes: [0, 2], (2, 4], (4, 6], (6, 8], and >8 m/s. To obtain a regional overview, a composite wind rose diagram for the Southern Yellow Sea was generated by aggregating all station data, with bars oriented to indicate the direction from which the wind originated. The plot was configured with a polar coordinate system (0° at north, clockwise orientation), stacked bars colored by wind speed class, and radial axes representing observation frequency.

## 3. Results

### 3.1. Distribution of Ulva Micropropagules in Surface Sediments of the Southern Yellow Sea in 2020

**Figure 2:**
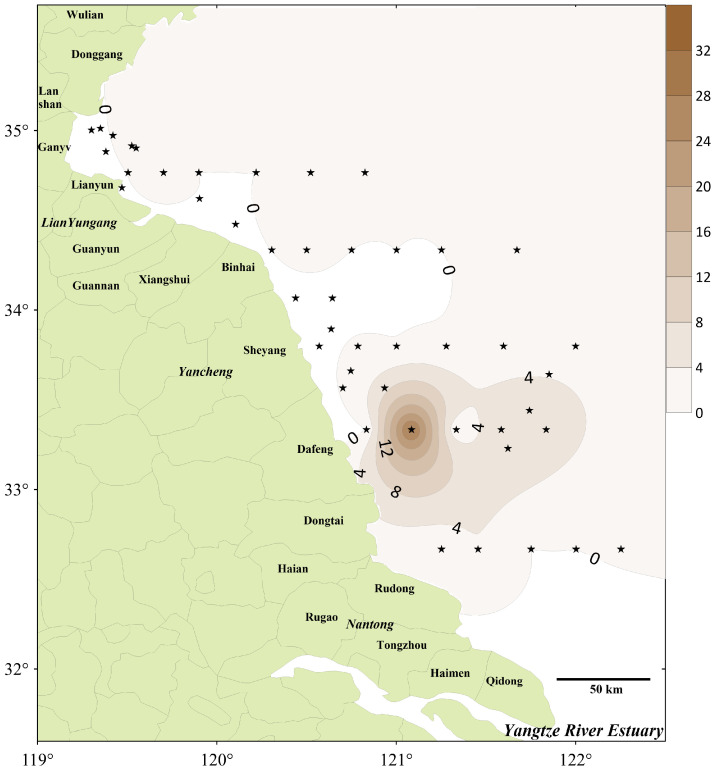
Micropropagule density in surface sediments in April 2020 (units: inds g^−1^). Note: Black stars represent stations; specific station numbers can be obtained from Figure 1. Meanwhile, the wind rose diagram aggregated from all stations for the corresponding sampling month is shown in Figure S2, and detailed information is provided in Table 1.

In April 2020 (before the large-scale outbreak of green tides in the Southern Yellow Sea), *Ulva* micropropagules were not detected in surface sediments at 11 stations (RD3, RD4, DF1, BH1, BH3–BH5, LYG1, LYG3–LYG5); small amounts of *Ulva* micropropagules were present in surface sediments at 8 stations (RD1, RD5, DF3, SY1–SY3, BH2, LYG2), with average abundances at each station all less than 1 inds g^−1^; *Ulva* micropropagules were present in surface sediments at 3 stations (RD2, DF4, DF5), with average abundances ranging from 4.0 to 7.6 inds g^−1^; the average abundance of *Ulva* micropropagules in surface sediments at station DF2 was the highest in that month, reaching 30.4 inds g^−1^ (Figure 1 and Figure 2). In that month, *Ulva* micropropagules in surface sediments were mainly concentrated at station DF2 and its vicinity (Figure 1 and Figure 2), while stations DF2 and RD2 are located in the laver aquaculture raft area (and its vicinity) of the Subei Shoal (Figure 1); the presence of *Ulva* micropropagules in surface sediments in this area is closely related to the Subei Shoal radial sand ridge area (the Subei Shoal has long been recognized as the *Ulva* “seed source” area); the presence of *Ulva* micropropagules in surface sediments at stations DF4 and DF5 (Figure 1 and Figure 2) may be related to the transport of *Ulva* micropropagules from the Subei Shoal area (possibly released by attached *Ulva* macroalgae in the Subei Shoal area, or possibly released by small-scale floating (or suspended) *Ulva* macroalgae in that area) to the open sea through tidal channels under the action of ocean current dynamics, with partial settling into surface sediments in that area; if released by small-scale floating (or suspended) *Ulva* macroalgae, since the accumulation of *Ulva* micropropagules in surface sediments from the water column is a gradual process, this implies that sporadic floating (or suspended) *Ulva* macroalgae had already been released and floated to the open sea before April.

**Figure 3:**
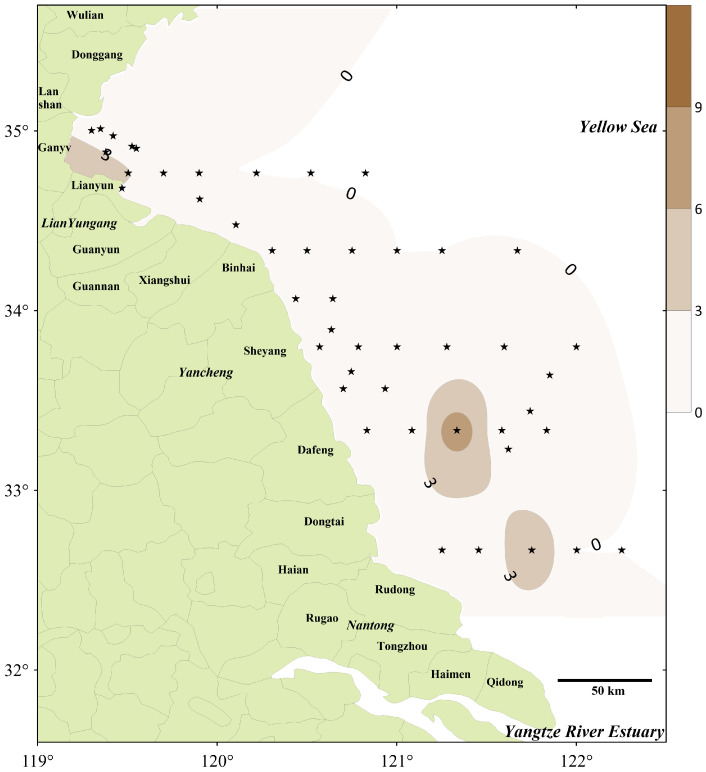
Micropropagule density in surface sediments in May 2020 (units: inds g^−1^). Note: Black stars represent stations; specific station numbers can be obtained from Figure 1. Meanwhile, the wind rose diagram aggregated from all stations for the corresponding sampling month is shown in Figure S3, and detailed information is provided in Table 1.

In May 2020 (during the large-scale outbreak of green tides in the Southern Yellow Sea), *Ulva* micropropagules were not detected in surface sediments at 12 stations (RD2, RD4, RD5, DF2, DF4, SY2, BH2, BH4, BH5, LYG3–LYG5); small amounts of *Ulva* micropropagules were present in surface sediments at 6 stations (DF1, DF5, SY1, BH1, BH3, LYG2), with average abundances all less than 1 inds g^−1^; *Ulva* micropropagules were present in surface sediments at 5 stations (RD1, RD3, DF3, SY3, LYG1), with average abundances ranging from 1.6 to 9.2 inds g^−1^. The average abundance of *Ulva* micropropagules in surface sediments at station DF3 was the highest in that month, reaching 9.2 inds g^−1^ (Figure 1 and Figure 3); station RD3 ranked second, with an average abundance of *Ulva* micropropagules in surface sediments of 6.0 inds g^−1^ (Figure 1 and Figure 3). Stations DF3 and RD3 are located near the Subei Shoal area (Figure 1); the presence of *Ulva* micropropagules in surface sediments at these stations may be related to the transport of *Ulva* micropropagules from the Subei Shoal area (possibly released by attached *Ulva* macroalgae in the Subei Shoal area, or possibly released by small-scale floating (or suspended) *Ulva* macroalgae in that area) to the open sea through tidal channels under the action of ocean current dynamics, with partial settling into surface sediments in that area. The presence of *Ulva* micropropagules in surface sediments at station LYG1 (Figure 1 and Figure 3), with an average abundance of 3.2 inds g^−1^, slightly lower than station RD3, may be related to the Haizhou Bay area also being a traditional laver aquaculture area (with attached *Ulva* macroalgae on aquaculture rafts).

In July 2020 (during the decline period of green tides in the Southern Yellow Sea), *Ulva* micropropagules were not found in surface sediments at any of the sampling stations (RD1–RD5, DF1–DF5, SY1–SY3, BH1–BH5, and LYG1–LYG5; the wind rose diagram aggregated from all stations for the corresponding sampling month is shown in Figure S4). This means that, within the scope of this study, after the large-scale green tide algae in the Southern Yellow Sea declined, the *Ulva* micropropagule germplasm transported to surface sediments in the open sea (e.g., south of the 35° N line) could hardly survive until the floating period of green tides in the following year.

### 3.2. Distribution of Ulva Micropropagules in Surface Sediments of the Southern Yellow Sea in 2021

**Figure 4:**
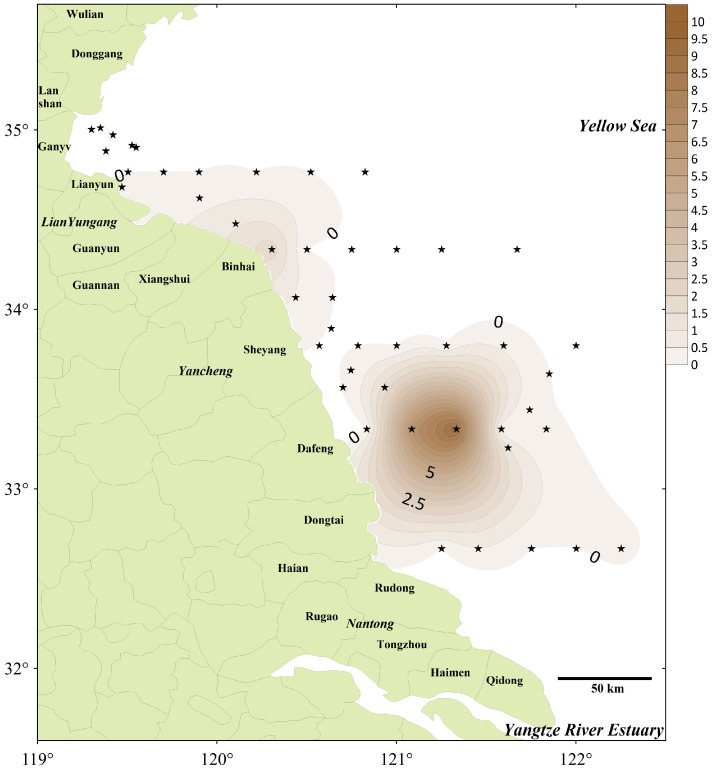
Micropropagule density in surface sediments in March 2021 (units: inds g^−1^). Note: Black stars represent stations; specific station numbers can be obtained from Figure 1. Meanwhile, the wind rose diagram aggregated from all stations for the corresponding sampling month is shown in Figure S5, and detailed information is provided in Table 1.

In March 2021 (before the large-scale outbreak of green tides in the Southern Yellow Sea), *Ulva* micropropagules were not detected in surface sediments at 15 stations (RD1, RD3, RD4, DF1, SY1, SY2, BH2–BH5, LYG1–LYG5); small amounts of *Ulva* micropropagules were present in surface sediments at 5 stations (RD2, RD5, DF4, DF5, SY3), with average abundances at each station ranging from 0.1 to 0.5 inds g^−1^; *Ulva* micropropagules were present in surface sediments at station BH1, with an average abundance of 1.8 inds g^−1^; the average abundance of *Ulva* micropropagules in surface sediments at stations DF2–DF3 was the highest in that month, ranging from 6.7 to 9.5 inds g^−1^ (Figure 1 and Figure 4). In that month, *Ulva* micropropagules in surface sediments were mainly concentrated at stations DF2, DF3, and their vicinity (Figure 1 and Figure 4), located in the laver aquaculture raft area (and its vicinity) of the Subei Shoal (Figure 1); the presence of *Ulva* micropropagules in surface sediments in this area is closely related to the Subei Shoal radial sand ridge area. *Ulva* micropropagules with abundance second only to the Subei Shoal area were also present in surface sediments at station BH1 (Figure 1 and Figure 4). Since in July 2020, *Ulva* micropropagules were not found in surface sediments at any sampling stations within the study area; this means that the *Ulva* micropropagules detected in March 2021 were most likely newly released to the sea area and settled into surface sediments between July 2020 and March 2021.

**Figure 5:**
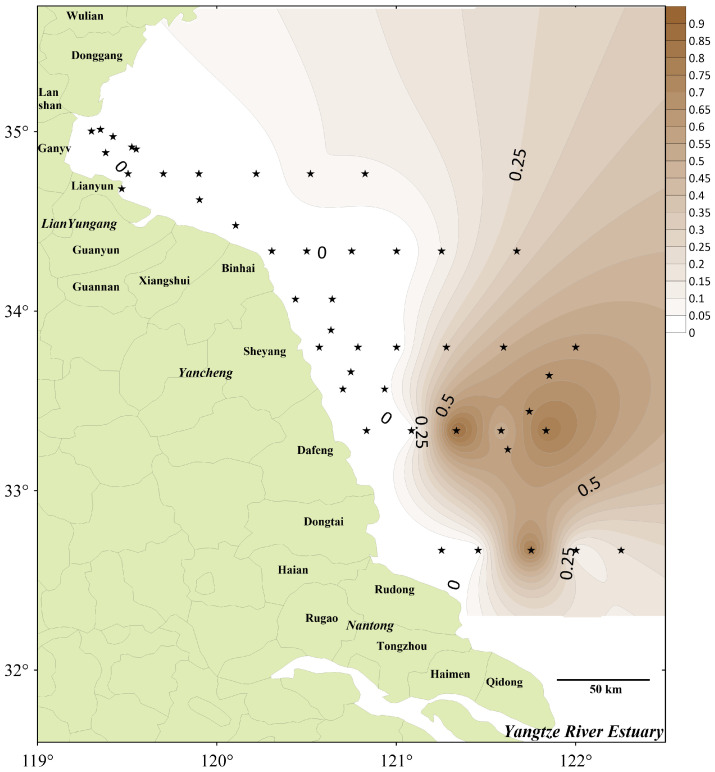
Micropropagule density in surface sediments in May 2021 (units: inds g^−1^). Note: Black stars represent stations; specific station numbers can be obtained from Figure 1. Meanwhile, the wind rose diagram aggregated from all stations for the corresponding sampling month is shown in Figure S6, and detailed information is provided in Table 1.

In May 2021 (during the large-scale outbreak of green tides in the Southern Yellow Sea), *Ulva* micropropagules were not detected in surface sediments at 14 stations (RD1, RD2, DF1, DF2, SY1, SY2, BH1–BH4, LYG1–LYG4); small amounts of *Ulva* micropropagules were present in surface sediments at 6 stations (RD4, RD5, DF4, SY3, BH5, LYG5), with average abundances at each station ranging from 0.1 to 0.5 inds g^−1^; the average abundance of *Ulva* micropropagules in surface sediments at stations RD3, DF3, and DF5 was the highest in that month, ranging from 0.8 to 0.9 inds g^−1^ (Figure 1 and Figure 5). In that month, *Ulva* micropropagules in surface sediments were mainly distributed in the sea area east of the Subei Shoal, located in the open sea, and the distribution range was much larger than the same period in 2020 (Figure 1, Figure 3 and Figure 5); this means that in May 2021, floating (or suspended) green tide algae had abnormally diffused to the open sea on a large scale, and *Ulva* micropropagules released by floating (or suspended) green tide algae had partially settled into surface sediments in that sea area.

**Figure 6:**
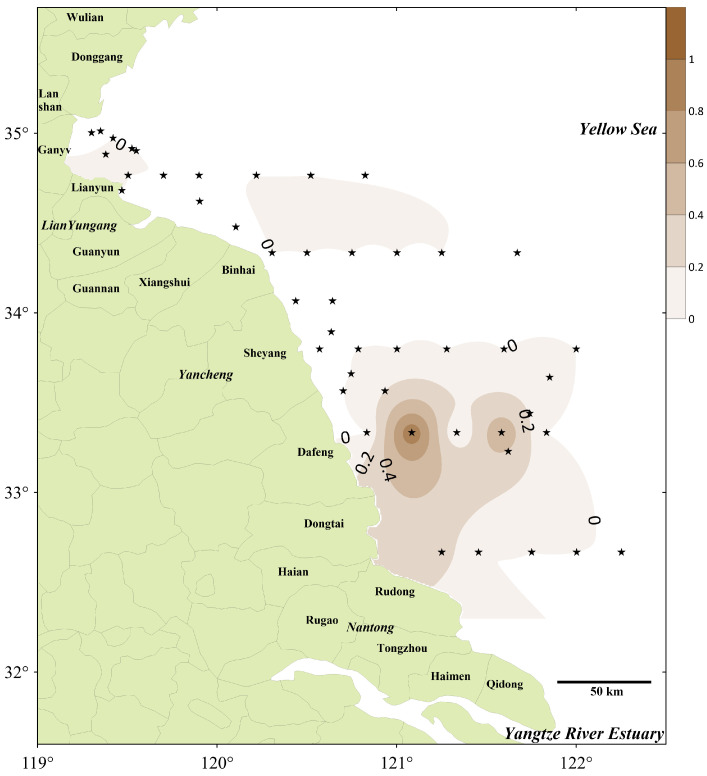
Micropropagule density in surface sediments in November 2021 (units: inds g^−1^). Meanwhile, the wind rose diagram aggregated from all stations for the corresponding sampling month is shown in Figure S7, and detailed information is provided in Table 1. Note: Black stars represent stations; specific station numbers can be obtained from Figure 1.

In November 2021 (after the complete decline of green tides in the Southern Yellow Sea), *Ulva* micropropagules were not detected in surface sediments at 29 stations (RD2–RD5, DF1, DF3, DF5, SY0, SY1-1, SY1-2, SY1–SY5, BH1–BH6, LYG1-1, LYG1-2, LYG1-3, LYG1–LYG5); small amounts of *Ulva* micropropagules were present in surface sediments at stations RD1 and DF4, with average abundances at each station ranging from 0.3 to 0.6 inds g^−1^ (Figure 1 and Figure 6). In that month, *Ulva* micropropagules in surface sediments were mainly concentrated at station DF2 (average abundance of 1.0 inds g^−1^) and its vicinity (Figure 1 and Figure 6), and the presence of *Ulva* micropropagules in surface sediments in this area is closely related to the Subei Shoal radial sand ridge area. Based on data from July 2020, this study found that after the large-scale green tide algae in the Southern Yellow Sea declined, the *Ulva* micropropagule germplasm transported to surface sediments in the open sea (e.g., south of the 35° N line) could hardly survive until the floating period of green tides in the following year; therefore, the *Ulva* micropropagules detected in surface sediments at individual stations within the study area in November 2021 were most likely newly released to the sea area and settled into surface sediments between the complete decline of the 2021 Southern Yellow Sea green tide and November 2021; and this phenomenon is closely related to the Subei Shoal radial sand ridge area (Figure 1 and Figure 6), at which time a new round of aquaculture activities was often being carried out in the laver aquaculture raft area of the Subei Shoal (the new laver aquaculture cycle was from the autumn of 2021 to the spring of 2022 in the Northern Hemisphere).

### 3.3. Distribution of Ulva Micropropagules in Surface Sediments of the Southern Yellow Sea in 2022

**Figure 7:**
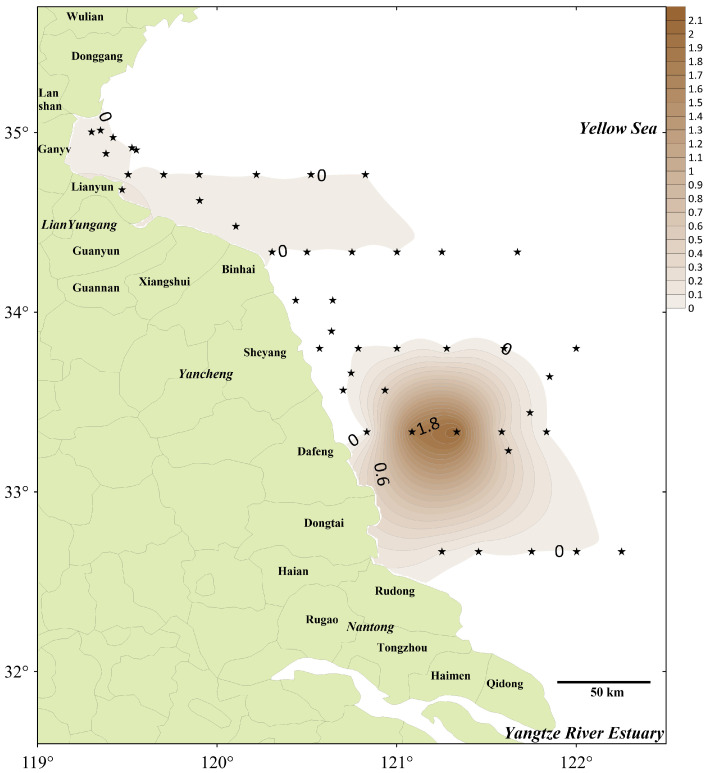
Micropropagule density in surface sediments in March 2022 (units: inds g^−1^). Note: Black stars represent stations; specific station numbers can be obtained from Figure 1. Meanwhile, the wind rose diagram aggregated from all stations for the corresponding sampling month is shown in Figure S8, and detailed information is provided in Table 1.

In March 2022 (before the large-scale outbreak of green tides in the Southern Yellow Sea), *Ulva* micropropagules were not detected in surface sediments at 26 stations (RD2–RD5, DF1, DF5, SY0–SY4, SY1-1, SY1-2, BH1–BH5, LYG1–LYG6, LYG1-2, LYG1-3); small amounts of *Ulva* micropropagules were present in surface sediments at 3 stations (RD1, DF4, LYG1-1), with average abundances at each station ranging from 0.1 to 0.4 inds g^−1^; the average abundance of *Ulva* micropropagules in surface sediments at stations DF2 and DF3 was the highest in that month, ranging from 1.8 to 2.1 inds g^−1^ (Figure 1 and Figure 7). In that month, *Ulva* micropropagules in surface sediments were mainly concentrated at stations DF2, DF3, and their vicinity (Figure 1 and Figure 7), located in the laver aquaculture raft area (and its vicinity) of the Subei Shoal (Figure 1).

**Figure 8:**
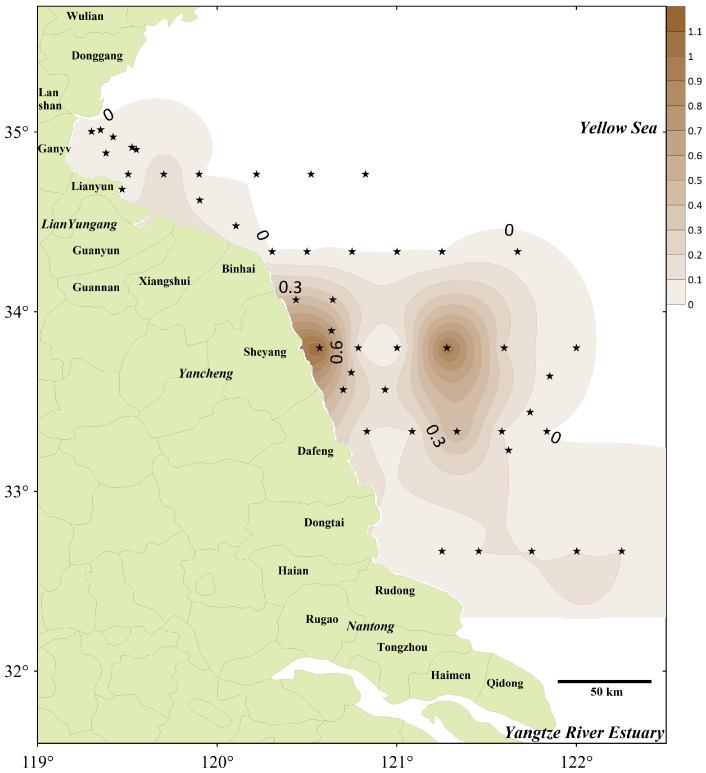
Micropropagule density in surface sediments in May 2022 (units: inds g^−1^). Note: Black stars represent stations; specific station numbers can be obtained from Figure 1. Meanwhile, the wind rose diagram aggregated from all stations for the corresponding sampling month is shown in Figure S9, and detailed information is provided in Table 1.

In May 2022 (during the large-scale outbreak of green tides in the Southern Yellow Sea), *Ulva* micropropagules were not detected in surface sediments at 12 stations (RD1, DF5, SY2, BH1–BH5, LYG1, LYG3–LYG5); small amounts of *Ulva* micropropagules were present in surface sediments at 11 stations (RD2–RD5, DF1–DF4, SY1, SY4, LYG2), with average abundances at each station ranging from 0.1 to 0.5 inds g^−1^; the average abundance of *Ulva* micropropagules in surface sediments at stations SY0 and SY3 was the highest in that month, ranging from 1.0 to 1.1 inds g^−1^ (Figure 1 and Figure 8). Within the study area, *Ulva* micropropagules in surface sediments in that month were mainly distributed in the sea area north of the Subei Shoal, with a distribution range smaller than the same period in 2021 (Figure 1, Figure 5 and Figure 8); this indicates that in that month, floating (or suspended) green tide algae had diffused to coastal waters on a considerable scale, and *Ulva* micropropagules released by floating (or suspended) green tide algae had partially settled into surface sediments in that sea area (Figure 8).

**Figure 9:**
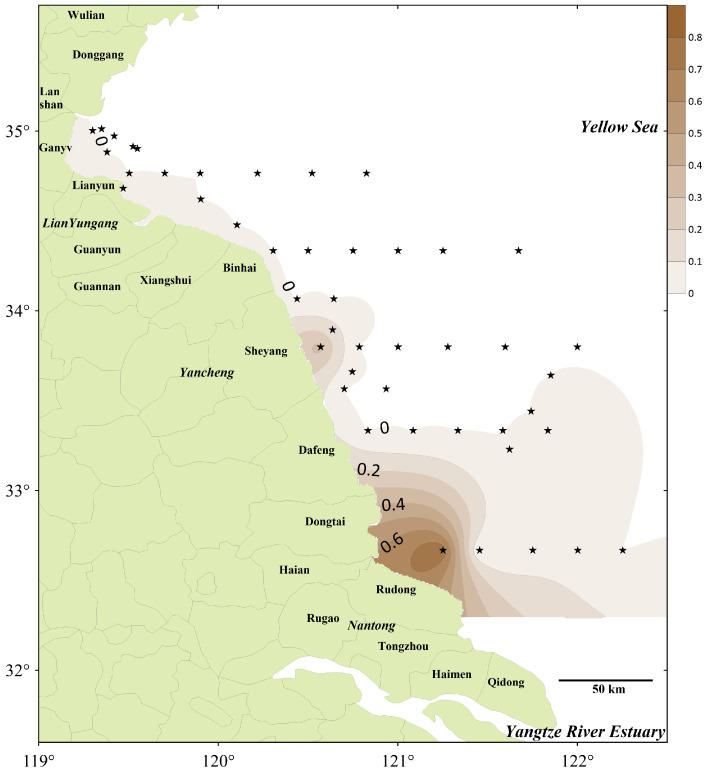
Micropropagule density in surface sediments in December 2022 (units: inds g^−1^). Note: Black stars represent stations; specific station numbers can be obtained from Figure 1. Meanwhile, the wind rose diagram aggregated from all stations for the corresponding sampling month is shown in Figure S10, and detailed information is provided in Table 1.

In December 2022 (after the complete decline of green tides in the Southern Yellow Sea), *Ulva* micropropagules were not detected in surface sediments at 33 stations (RD2, RD5, DF1–DF4, SY1–SY4, BH1–BH5, LYG1–LYG5, HZW1–HZW4, T1-1, T1-2, T2-1, T2-2, T3-1, T3-2, T3-3, T4-1, T4-2); small amounts of *Ulva* micropropagules were present in surface sediments at stations RD3, RD4, and DF5, with an average abundance of approximately 0.06 inds g^−1^; some *Ulva* micropropagules were also present in surface sediments at station SY0, with an average abundance of approximately 0.33 inds g^−1^ (Figure 1 and Figure 9). In that month, *Ulva* micropropagules in surface sediments were mainly concentrated at station RD1 (average abundance of 0.8 inds g^−1^) and its vicinity (Figure 1 and Figure 9); the presence of *Ulva* micropropagules in surface sediments in this area is closely related to the Subei Shoal radial sand ridge area; they were most likely newly released during a new round of aquaculture activities in the laver aquaculture raft area of the Subei Shoal (the new laver aquaculture cycle was from the autumn of 2022 to the spring of 2023 in the Northern Hemisphere), and had partially settled into surface sediments in that sea area.

## 4. Discussion

### 4.1. Spatiotemporal Distribution Characteristics of Ulva Micropropagules in Surface Sediments of the Southern Yellow Sea and Their Association with the Green Tide Outbreak Process

During green tide outbreaks in the Southern Yellow Sea, the species composition of floating algal communities shows obvious temporal succession, with multiple *Ulva* species often appearing successively or concurrently; in view of this, comprehensive consideration at the genus level for this group helps to more systematically understand the formation and evolution mechanisms of green tides in the Southern Yellow Sea [27,41,42,43]. In the surface sediment samples from the Southern Yellow Sea involved in this study, the abundance of *Ulva* micropropagules was generally not high, and showed significant heterogeneity in temporal and spatial dimensions. The spatial distribution pattern was always closely related to the laver aquaculture area in the radial sand ridges of the Subei Shoal: before large-scale green tide outbreaks, high-abundance areas were mainly concentrated in the sea area adjacent to the aquaculture rafts of the Subei Shoal; during large-scale green tide outbreaks, the distribution range may be regulated by the diffusion pattern of floating green tide algae, with obvious differences between different years, expanding to the open sea in large-scale green tide years and mainly limited to coastal waters in small-scale years; during (and after) the decline period, stations in the open sea were generally not detected, and only trace amounts of *Ulva* micropropagules remained in areas near the coast and the aquaculture area.

The monthly variation in *Ulva* micropropagule density has a potential temporal association with the green tide outbreak process. The relative enrichment before the outbreak may reflect the continuous and cumulative process of source release from the laver raft aquaculture area; the distribution in the open sea during the outbreak period may be attributed to release and settling accompanying the diffusion process of floating green tide algae; and the general disappearance after decline implies that the germplasm settled in the open sea is difficult to survive across years. However, the above inferences are based only on observations south of the 35° N line; for *Ulva* micropropagules settled north of this line, as well as large amounts of *Ulva* macroalgae settled in the coastal waters of Shandong that may not have completely died, their overwintering survival potential and germplasm contribution to the following year’s green tide remain unclear.

### 4.2. Exceptional Patterns in the Surface Sedimentary Micropropagule Record and Their Link to Interannual Green Tide Variability

While Section 4.1 describes the general spatiotemporal pattern of *Ulva* micropropagule distribution in relation to green tide dynamics, observations at some specific stations reveal notable deviations from this general framework, potentially reflecting the influence of supplementary sources, extreme weather events, and anthropogenic activities on the sedimentary micropropagule record.

The date when green tides were first detected by satellite remote sensing imagery was 21 May 2020, and the date when green tides disappeared from satellite remote sensing imagery was July 23 (the maximum coverage area reached its peak in June, at 192 km^2^) [21]. Notably, *Ulva* micropropagules with abundance second only to the Subei Shoal area were detected in surface sediments at station BH1 (Figure 1 and Figure 4), which is located outside the core laver aquaculture zone of the Subei Shoal. This finding appears to deviate from the general spatial pattern described in Section 4.1 and may suggest that the Binhai County to Sheyang County area of Yancheng City could have served as a supplementary source of green tide algae in March 2021 [17,18,27], potentially contributing micropropagules to regions beyond the primary source area.

The date when green tides were first detected by satellite remote sensing imagery was 18 May 2021, and the date when green tides disappeared from satellite remote sensing imagery was 18 August [21]. In May 2021, floating (or suspended) green tide algae had abnormally diffused to the open sea on a large scale (Figure 5). The scale of the 2021 Yellow Sea green tide outbreak was historically rare, with its maximum coverage area reaching a historical peak in June 2021 (1746 km^2^) [21]; when sporadic floating (or suspended) green tide algae mainly floated near coastal waters, the scale of the green tide outbreak that year tended to be small; when sporadic floating (or suspended) green tide algae diffused to the open sea prematurely, the scale of the green tide outbreak that year tended to be large. This abnormal rapid diffusion of floating green tide algae in 2021 was very likely related to extreme weather in the sea area near Jiangsu Province at the end of April 2021: on 30 April 2021, the coastal area of Jiangsu Province suffered rare extreme strong winds (maximum wind speed reaching Force 13) and thunderstorms, causing damage to some laver aquaculture rafts, and a large number of ropes, bamboo poles, and other aquaculture facilities, as well as green tide algae, were swept into the sea and rapidly transported to the open sea under the action of wind and ocean currents [21].

The date when green tides were first detected by satellite remote sensing imagery was 16 May 2020, and the date when green tides disappeared from satellite remote sensing imagery was 9 August, with the maximum coverage area of the 2022 Southern Yellow Sea green tide being approximately 135 km^2^ (reaching its peak in June). In December 2022, *Ulva* micropropagules were present in surface sediments at station SY0 (Figure 1 and Figure 9); however, in November 2021 (Figure 1 and Figure 6) and March 2022 (Figure 1 and Figure 7), *Ulva* micropropagules were not detected in surface sediments at station SY0. This temporal pattern constitutes a marked exception to the general pattern described in Section 4.1. There are mainly three possible explanations for this situation: first, the 2022 Yellow Sea green tide algae mainly floated near the coast and once appeared in large quantities near station SY0 (Figure 1 and Figure 8); due to the large scale of green tide algae accumulation or settling in that area, until December 2022, the green tide algae may not have completely died, and some green tide algae may still have been releasing small-scale *Ulva* micropropagules that entered surface sediments. Second, the Yellow Sea green tide algae that floated near coastal waters and once appeared in large quantities near station SY0 in 2022 may have released *Ulva* micropropagules that had already attached and grown in the sea area near station SY0 or its coast, and the newly formed *Ulva* macroalgae would release a certain scale of *Ulva* micropropagules during their growth process. Third, December to the following spring is an important period for cleaning coastal mariculture ponds; farmers generally drain and clean ponds and disinfect them after harvesting aquatic animals; a large number of coastal mariculture ponds exist in Sheyang County, Yancheng City, and *Ulva* macroalgae are present in these ponds and will release corresponding *Ulva* micropropagules [16]; during the pond cleaning process, farmers may have directly or indirectly caused *Ulva* micropropagules to be released to coastal waters and partially settle, resulting in the detection of a certain scale of *Ulva* micropropagules in surface sediments at station SY0.

### 4.3. Driving Mechanisms of Overwintering and Spring Proliferation of Ulva Micropropagules

At present, it is widely accepted among researchers that *Ulva* micropropagules within the sediments of the Subei Shoal constitute a critical link in the initiation of the Southern Yellow Sea green tide [30,44,45,46,47,48,49]. However, existing evidence remains insufficient to identify them as the sole direct trigger of the green tide outbreak, as the regional eutrophic background and distinctive mariculture practices also serve as significant drivers [50,51,52,53,54,55,56,57,58]. The specific mechanisms underlying the occurrence and development of the green tide can be described as follows: A portion of the *Ulva* micropropagules in the sediments survive the low-temperature conditions of winter [31,44]. Following the spring rise in water temperature, these micropropagules, under the regulation of biological rhythms (i.e., periodic changes in life activities driven by endogenous circadian clocks [59,60,61,62,63,64]; although current research on the circadian clock mechanism in *Ulva* micropropagules remains relatively limited and warrants further investigation), seek out suitable substrates for germination and growth. Whether biomagnetism [65,66,67,68] exerts any influence on this process remains unclear; for instance, it is presently unknown whether the geomagnetic field over the Subei Shoal or local magnetic fields generated by artificial structures (e.g., offshore wind turbine foundations, coastal dikes, artificial islands, and aquaculture operation vessels) affect the attachment orientation and germination efficiency of the micropropagules. Given the abundance of artificial structures in the Subei Shoal area, such as laver aquaculture rafts, wind turbine foundations, coastal dikes, artificial islands, and aquaculture vessels [69,70,71], these provide suitable attachment substrata for *Ulva* micropropagules, thereby acting as significant “amplifiers” that drive the continuous expansion of the background *Ulva* biomass that fuels green tide outbreaks [45,51,71,72].

Nevertheless, while current research focuses on the background abundance of *Ulva* micropropagules in the marine environment (e.g., in sediments), we propose two hypothetical mechanisms that warrant future investigation. It should be emphasized that these mechanisms remain speculative and lack direct experimental confirmation in the present study; they are presented here to guide future targeted research.

First, the magnitude of *Ulva* micropropagules discharged into coastal waters via drainage channels or river outlets from coastal aquaculture ponds in spring is considerable. Within these ponds, substantial quantities of epiphytic or free-floating nuisance *Ulva* algae are commonly present [16,19,20,73,74], which continuously release micropropagules into the pond water during their growth. Moreover, given the proximity of these coastal aquaculture ponds to the Subei Shoal area, frequent seawater exchange transports a portion of these micropropagules to the nearshore zone. Once these allochthonous micropropagules encounter suitable attachment substrates, they may initiate seasonal growth, thereby serving as an important supplementary source of epiphytic nuisance algae on artificial structures in the Subei Shoal.

Second, *Ulva* micropropagules that successfully attach to substrates continuously release new micropropagules during their growth. These newly released propagules follow two fates: some remain and colonize the original substrate, while others enter the water column or sediment environment and remain viable for a certain period. It is important to clarify that these newly generated micropropagules are not residual background propagules from the previous year, but rather “fresh” seed stock produced by the current growing generation. This self-proliferation mechanism implies that, even if the initial inoculum originates entirely from external sources (e.g., discharges from aquaculture ponds), once a colonizing population becomes established on artificial structures, exponential biomass expansion can occur through sustained propagule release, thereby significantly amplifying the potential risk of a green tide outbreak.

### 4.4. The Data from This Study Have Guiding Significance but Still Have Room for Research Improvement

This study focuses on surface sediments in the Southern Yellow Sea from 2020 to 2022, systematically revealing the spatiotemporal distribution characteristics of *Ulva* micropropagules in the study area, and exploring the potential associations between their monthly density variation patterns and the green tide outbreak process. Objectively speaking, the data from this study have important guiding and practical value for existing scientific hypotheses and subsequent research, but the following areas for improvement still exist: first, limited by the division of sea area and institutional responsibilities for marine surveys, this survey mainly covered the sea area south of 35° N; in the future, survey and research resources need to be integrated to include Shandong coastal stations into a unified research framework, to improve the understanding of *Ulva* micropropagule distribution at the regional scale; second, although large-area station surveys can grasp the overall situation of the sea area, the distribution characteristics of micropropagules in special habitats such as intertidal wetlands, estuarine channels, and mariculture pond discharge outlets may exhibit spatial heterogeneity, and the current survey network is still difficult to fully capture the special cases of these key “hotspot” areas; third, *Ulva* micropropagules may have different selective attachment preferences for sediment substrates of different materials (such as siliceous sand, calcareous sand, organic detritus), mineral composition proportions, and particle size distributions [75,76,77,78,79], and this substrate–propagule interaction mechanism may directly constrain the settling, burial, and resuspension processes of micropropagules in sediments, which is a direction that needs to be focused on and deepened in future surface sediment survey research; fourth, although previous studies have collected suspended *Ulva* macroalgae in coastal and radial sand ridge sea areas of the Southern Yellow Sea in March [29], their source attribution remains unclear, whether originating from detachment of laver aquaculture rafts, discharge from mariculture ponds, or natural detachment from intertidal habitats [11,18,19,80,81]; this source tracing issue is directly related to the weight evaluation of the “raft-origin-hypothesis” and the “micropropagule-hypothesis”, and in the future, comprehensive analysis urgently needs to be conducted combining molecular source tracing markers (such as microsatellite DNA or SNP genotyping [82,83,84]) and drift trajectory simulation techniques [85,86], to clarify the relative contribution rates of *Ulva* macroalgae from different sources to green tide outbreaks.

### 4.5. The Value of Laboratory Simulation Experiments and the Necessity of Their Integration with Field Surveys

In recent years, laboratory experiments have systematically analyzed the growth adaptability of *Ulva* macroalgae under environmental factor gradients such as temperature, light, salinity, and pH [87,88,89,90,91,92,93], and regarding the environmental response mechanisms of *Ulva* micropropagule germination and development, although related research started relatively late, it has gradually gained academic attention and accumulated preliminary results [94,95,96,97,98,99,100]. However, to establish “micropropagules in seabed sediments can survive for a long time (e.g., in the open sea) and directly drive green tide outbreaks in the following year” as a verifiable scientific hypothesis, at least the following four prerequisite conditions need to be met:

First, germination feasibility in low-light/low-temperature environments. The seabed sediment environment is characterized by weak light (even complete darkness) and temperatures significantly lower than surface water. Whether *Ulva* micropropagules can complete germination and maintain growth under such conditions currently lacks direct evidence, and direct experimental verification is needed.

Second, physiological tolerance in high-pressure environments. Unlike indoor atmospheric pressure experimental conditions, the seabed habitat has significant water pressure gradients. Given that *Ulva* micropropagules often germinate and grow in intertidal and coastal waters [101,102], their physiological tolerance and germination activity in offshore high-pressure environments are still unknown.

Third, survival and activity maintenance after biological disturbance. Under the action of biological disturbance processes such as marine organism feeding and microbial degradation [31,45,103,104,105,106,107,108,109], after a certain period of time, the residual proportion of micropropagules formed from settled green tide algae in sediments and the degree of biological activity maintenance are directly related to the actual contribution potential of the “seed source”.

Fourth, ecological fate pathways after germination. Whether the germination products of *Ulva* micropropagules in sediments can directly detach from the substrate environment and enter the water body to continue suspended growth, or must undergo a benthic growth stage before converting to a floating ecotype, the presence of micropropagules in sediments does not necessarily equate to the triggering of green tide outbreaks; the potential pathways for their conversion to floating algae and possible environmental triggering mechanisms need to be verified combining sea area field surveys and seabed in situ observation techniques.

Existing research has not yet provided clear answers to the above four core questions. Current laboratory experiments mostly adopt single-factor or multi-factor control designs [31,110], and their environmental parameter settings differ from actual seabed habitats, limiting the extrapolation of experimental results. Therefore, it is recommended that while continuing laboratory simulation experiments, vigorous efforts should be made to advance sea area field surveys (including in situ settling simulation experiments, routine monitoring during seabed observation processes, etc.), through indoor-field collaborative verification, to effectively clarify whether micropropagules have a conversion mechanism from “potential source” to “direct contribution”.

### 4.6. Future Research Needs to Conduct More Systematic Stratification and Chronology Studies on Sediment Samples

Currently, the sediment samples obtained by this study using benthic corers only represent surface sediment information at specific time points, making it difficult to reveal the historical distribution patterns of *Ulva* micropropagules in the vertical direction and their association with interannual variations in green tide outbreaks. Future research needs to introduce gravity coring techniques [111,112], to obtain continuous and complete sediment core sequences, and combine radionuclide dating methods such as ^210^Pb and ^137^Cs for precise dating of sediments [113,114,115], to establish a high-resolution time scale. On this basis, systematic processing of sediment samples from different depth layers, combined with recovery culture experiments, will quantitatively assess the abundance, viability, and germination potential of *Ulva* micropropagules in each layer. This method will help reconstruct the historical evolution sequence of *Ulva* algal population dynamics in the study area, clarify the formation timing and accumulation process of the potential “seed source” on the seabed, and thereby further deeply verify the potential hypothesis that “micropropagules settled over the years may drive green tide outbreaks”, and provide more solid data support for assessing the long-term ecological effects of green tide disasters and predicting future outbreak risks.

However, achieving the above research vision faces significant methodological challenges and resource limitations. Gravity coring and subsequent isotope dating and stratified culture analysis require high cost investment and long analysis cycles, and the broad sea area and numerous stations covered by this study (if all implemented) will further amplify this demand. Limited by sampling ship time, operation cycles, and budget constraints, systematic sediment core sampling at all stations is not currently feasible. In fact, stratified sediment research related to *Ulva* micropropagules is still relatively scarce. Although Liu et al. [116] conducted sediment stratified sampling (surface layer, 5 cm, 9 cm, 13 cm, 17 cm) in the Subei Shoal area in September and November 2017, and found through culture experiments that green algal micropropagule abundance decreased with increasing depth, similar studies are not only few in number but also rarely combined with isotope dating techniques to establish a chronological framework. Therefore, future research should adopt a phased, regional strategy: prioritize high-resolution sediment core analysis at key sections or representative stations with stable sedimentary environments and significant historical green tide impacts, to establish methodological paradigms; meanwhile, special funding support and cross-institutional technical cooperation need to be sought, to gradually advance large-scale, long-sequence verification and research of sediment “seed sources”.

## 5. Conclusions

Through systematic sediment sampling and laboratory culture experiments in the Southern Yellow Sea for three consecutive years from 2020 to 2022, this study revealed the spatiotemporal distribution characteristics of *Ulva* micropropagules in surface sediments and their association patterns with the green tide outbreak process. The results showed that the abundance of *Ulva* micropropagules was generally low and spatially heterogeneous, with high-value areas always closely related to the laver aquaculture raft area of the Subei Shoal; *Ulva* micropropagules diffused to the open sea during green tide outbreaks, but generally disappeared in the open sea after the decline period, making it difficult to form cross-year germplasm reserves. This finding does not support the “micropropagule-hypothesis” that “the germplasm bank in open sea sediments drives green tide outbreaks in the following year”, but instead strengthens the key role of the laver aquaculture raft area as a continuous source release zone.

However, this study still has limitations: for example, the survey range mainly covered the sea area south of 35° N, and special habitats such as the coastal waters of Shandong, intertidal zones, and mariculture pond discharge outlets were not fully included. While our findings indicate that the abundance of *Ulva* micropropagules in sediments was generally low and spatially linked to the laver aquaculture area, we acknowledge that additional unsampled habitats, environmental processes, or ecological mechanisms may also contribute to green tide dynamics. Future research should prioritize advancing collaborative verification between laboratory simulations and sea area field surveys, focusing on analyzing the germination feasibility, high-pressure tolerance, and pathway mechanisms for conversion to floating ecotypes of *Ulva* micropropagules in weak-light (dark) and low-temperature environments on the seabed, while expanding monitoring coverage to include the aforementioned unsampled habitats to more comprehensively evaluate potential source contributions. Moreover, seizing opportunities to conduct sediment core sampling and precise dating at key stations would help establish a high-resolution historical evolution sequence of green tide sources, and provide a more solid scientific basis for the precise prevention and control of green tide disasters and the establishment of early warning systems in the Southern Yellow Sea.

Beyond the Southern Yellow Sea, the role of benthic micropropagule banks in driving recurrent green tides remains a globally relevant question. In coastal bays and eutrophic embayments elsewhere, sediment-resident micropropagules have been proposed as critical overwintering reservoirs, yet whether such mechanisms operate in expansive shelf seas has rarely been tested at the field scale. We therefore encourage parallel field investigations in other coastal regions, particularly those with large-scale aquaculture or pronounced eutrophication, to assess whether the patterns observed here reflect a broader principle for open-shelf systems. A globally coordinated, standardized approach to sediment micropropagule monitoring would ultimately enable cross-regional validation of the “micropropagule-hypothesis” and refine predictive frameworks for green tide risk assessment worldwide.

## Figures and Tables

**Table 1:** Bottom water depth, wind speed, and wind direction during surface sediment sampling at 46 fixed and temporary monitoring stations. Sampling was conducted in April, May, and July 2020; March, May, and November 2021; and March, May, and December 2022.

Station Type	Sampling Station	Sampling Date (Year-Month)	Bottom Water Depth During Surface Sediment Sampling (m)	Wind Speed (m/s)	Wind Direction (16 Sectors)
Fixed Monitoring Station	RD1	April 2020	14	7.3	E
		May 2020	18.1	3.3	W
		July 2020	21.5	3.8	S
		March 2021	21	4.2	W
		May 2021	17.5	4.5	ESE
		November 2021	19.5	4.4	NE
		March 2022	17.5	5.7	S
		May 2022	3	3.1	WSW
		December 2022	17.3	4.6	NW
Fixed Monitoring Station	RD2	April 2020	22	8.9	E
		May 2020	19.3	2.5	W
		July 2020	22	3.5	SW
		March 2021	21	4.3	W
		May 2021	14.1	5.2	SE
		November 2021	23	4.5	NNE
		March 2022	22	3.4	NW
		May 2022	3.2	3.0	WSW
		December 2022	16.8	3.1	E
Fixed Monitoring Station	RD3	April 2020	18.7	5.9	NNE
		May 2020	8.7	4.5	NW
		July 2020	11	2.9	SW
		March 2021	15	5.2	W
		May 2021	9.8	2.3	S
		November 2021	11	5.6	ENE
		March 2022	12	2.7	W
		May 2022	7.8	2.0	WSW
		December 2022	14.1	3.4	SSE
Fixed Monitoring Station	RD4	April 2020	19	4.3	NE
		May 2020	17.8	4.0	NNW
		July 2020	20	2.7	SW
		March 2021	19	5.0	W
		May 2021	20.6	1.9	S
		November 2021	20	5.7	ENE
		March 2022	19	6.9	W
		May 2022	9.6	2.0	E
		December 2022	20.4	3.4	ENE
Fixed Monitoring Station	RD5	April 2020	27	6.5	ENE
		May 2020	28.2	4.0	NE
		July 2020	29.5	3.2	S
		March 2021	31	5.6	W
		May 2021	30.8	3.1	SE
		November 2021	31	3.9	ENE
		March 2022	33	6.1	NNE
		May 2022	12.7	1.5	E
		December 2022	12	4.1	NE
Fixed Monitoring Station	DF1	April 2020	24	1.0	NNE
		May 2020	18.5	2.5	ESE
		July 2020	16	6.0	SE
		March 2021	22	0.7	NE
		May 2021	18.2	5.2	SE
		November 2021	20	6.8	ENE
		March 2022	16.5	5.5	S
		May 2022	16	3.0	SSW
		December 2022	13.6	4.6	NNE
Fixed Monitoring Station	DF2	April 2020	10	1.0	NNE
		May 2020	11	2.8	ESE
		July 2020	18	7.0	SW
		March 2021	9	1.0	WNW
		May 2021	9.1	2.0	SSE
		November 2021	13.8	4.6	ESE
		March 2022	13	7.6	SSE
		May 2022	18.6	6.5	SSW
		December 2022	16	6.0	NW
Fixed Monitoring Station	DF3	April 2020	14	3.8	NE
		May 2020	10	1.2	SE
		July 2020	12	4.5	SW
		March 2021	9	2.4	SE
		May 2021	7.3	2.1	SSE
		November 2021	14.7	3.1	N
		March 2022	8.5	8.6	ENE
		May 2022	7	3.5	SSW
		December 2022	13	7.2	ESE
Fixed Monitoring Station	DF4	April 2020	13	3.5	N
		May 2020	15	1.9	SE
		July 2020	11.5	5.4	SW
		March 2021	15	7.9	SE
		May 2021	14.6	2.8	SE
		November 2021	19	5.2	ESE
		March 2022	16	5.1	ENE
		May 2022	13	3.0	SSW
		December 2022	19	8.9	NW
Fixed Monitoring Station	DF5	April 2020	8	4.3	NE
		May 2020	12.7	2.4	SE
		July 2020	13.5	5.3	W
		March 2021	14	4.9	S
		May 2021	13.2	1.6	SSE
		November 2021	15	6.8	E
		March 2022	14	5.8	SE
		May 2022	11	3.6	W
		December 2022	18.2	4.5	WNW
Temporary Monitoring Station	SY0	November 2021	8	1.9	SSE
		March 2022	5	6.1	SW
		May 2022	5	3.6	E
		December 2022	15.6	2.3	W
Fixed Monitoring Station	SY1	April 2020	15	1.2	NNE
		May 2020	15	0	SSW
		July 2020	16.5	3.4	SW
		March 2021	19	6.1	SE
		May 2021	17	6.4	SSE
		November 2021	17.8	1.5	SSW
		March 2022	18	4.1	S
		May 2022	19	3.0	E
		December 2022	19.2	5.5	SSE
Temporary Monitoring Station	SY1-1	November 2021	17	5.1	S
		March 2022	17	5.2	S
Temporary Monitoring Station	SY1-2	November 2021	15	2.6	SE
		March 2022	13.5	6.2	E
Fixed Monitoring Station	SY2	April 2020	14	1.8	NE
		May 2020	17	4.3	ENE
		July 2020	16	2.7	W
		March 2021	18	4.5	SE
		May 2021	16	2.8	SSW
		November 2021	18.5	6.2	W
		March 2022	18.5	5.7	SE
		May 2022	17.6	3.3	E
		December 2022	16.8	3.2	ENE
Fixed Monitoring Station	SY3	April 2020	13	2.5	NNE
		May 2020	19	3.6	ESE
		July 2020	16	1.7	SW
		March 2021	17	4.2	SE
		May 2021	17	5.2	SSW
		November 2021	17	5.6	W
		March 2022	17	1.9	SSE
		May 2022	15	4.0	E
		December 2022	17.5	8.3	SSE
Temporary Monitoring Station	SY4	November 2021	17.5	6.0	SW
		March 2022	19	2.0	ESE
		May 2022	17.3	4.5	E
		December 2022	7	7.8	NE
Temporary Monitoring Station	SY5	November 2021	16	7.1	W
Fixed Monitoring Station	BH1	April 2020	13	2.5	N
		May 2020	7	3.7	SSE
		July 2020	17	4.7	W
		March 2021	7	2.5	NW
		May 2021	10	1.4	SW
		November 2021	18	4.1	N
		March 2022	17	5.5	E
		May 2022	16	2.0	E
		December 2022	18.6	6.9	N
Fixed Monitoring Station	BH2	April 2020	16	4.0	N
		May 2020	17.5	1.4	W
		July 2020	18.5	4.5	SW
		March 2021	18	4.1	SW
		May 2021	20	2.2	SW
		November 2021	17.8	4.7	WNW
		March 2022	19	5.8	S
		May 2022	19	2.5	ESE
		December 2022	10.6	5.1	NE
Fixed Monitoring Station	BH3	April 2020	16	4.5	S
		May 2020	18	1.5	S
		July 2020	19	4.1	W
		March 2021	19	3.5	SSW
		May 2021	16	2.5	S
		November 2021	18.3	4.0	W
		March 2022	19.5	5.4	SSW
		May 2022	19.7	2.5	SSE
		December 2022	17.2	4.9	NW
Fixed Monitoring Station	BH4	April 2020	17	2.0	NNW
		May 2020	18.5	4.0	NNE
		July 2020	19	5.3	SW
		March 2021	19	4.6	SSW
		May 2021	20	4.0	SSW
		November 2021	19.5	6.6	WNW
		March 2022	19.5	3.9	S
		May 2022	19.7	4.0	SSE
		December 2022	18	9.9	SE
Fixed Monitoring Station	BH5	April 2020	18	3.0	NE
		May 2020	20	3.7	E
		July 2020	18.5	3.4	SW
		March 2021	20	4.7	SSW
		May 2021	21	4.5	S
		November 2021	20.5	4.3	WNW
		March 2022	23	5.7	S
		May 2022	20	4.0	SSE
		December 2022	12.2	2.7	NNE
Temporary Monitoring Station	BH6	November 2021	19.5	6.1	NNW
Fixed Monitoring Station	LYG1	April 2020	6	5.2	NNE
		May 2020	8	3.7	E
		July 2020	11.5	6.4	SW
		March 2021	9	2.9	ESE
		May 2021	6	2.2	SSE
		November 2021	9.5	1.5	ENE
		March 2022	9.5	3.3	ENE
		May 2022	12	3.5	S
		December 2022	18.3	5.7	SSE
Temporary Monitoring Station	LYG1-1	November 2021	6.5	1.8	E
		March 2022	4.5	7.4	E
Temporary Monitoring Station	LYG1-2	November 2021	18.5	5.6	S
		March 2022	19	3.2	SSW
Temporary Monitoring Station	LYG1-3	November 2021	14	3.2	SSW
		March 2022	17	4.3	ESE
Fixed Monitoring Station	LYG2	April 2020	11	4.8	NNE
		May 2020	14	4.8	SE
		July 2020	15.5	4.9	SW
		March 2021	8	4.1	ESE
		May 2021	12	1.1	WNW
		November 2021	11.5	1.1	E
		March 2022	16	7.1	E
		May 2022	14	3.5	SSE
		December 2022	18.8	4.7	S
Fixed Monitoring Station	LYG3	April 2020	17	3.8	NNE
		May 2020	17	5.1	SSE
		July 2020	16	4.1	SW
		March 2021	17	2.2	ESE
		May 2021	16	4.6	ESE
		November 2021	16	1.9	NW
		March 2022	18.5	9.2	ENE
		May 2022	16.5	6.0	S
		December 2022	14.3	2.7	NE
Fixed Monitoring Station	LYG4	April 2020	18	4.0	NNE
		May 2020	20	4.3	SSE
		July 2020	17.5	3.3	W
		March 2021	23	1.3	E
		May 2021	17	3.6	E
		November 2021	17.5	4.2	SW
		March 2022	18.6	7.2	ENE
		May 2022	18	6.3	SSW
		December 2022	19.6	6.8	SW
Fixed Monitoring Station	LYG5	April 2020	22	4.3	N
		May 2020	26	3.0	S
		July 2020	24	2.5	SW
		March 2021	26	0.5	E
		May 2021	24.8	2.3	ENE
		November 2021	24	5.5	ENE
		March 2022	25	5.3	E
		May 2022	22	6.0	SW
		December 2022	9.2	2.0	E
Temporary Monitoring Station	LYG6	March 2022	28.5	6.2	ESE
Temporary Monitoring Station	HZW1	December 2022	9.7	4.0	ENE
Temporary Monitoring Station	HZW2	December 2022	8	5.1	ESE
Temporary Monitoring Station	HZW3	December 2022	25.5	5.9	ESE
Temporary Monitoring Station	HZW4	December 2022	9	1.8	S
Temporary Monitoring Station	T1-1	December 2022	23	3.5	SE
Temporary Monitoring Station	T1-2	December 2022	19.6	2.7	S
Temporary Monitoring Station	T2-1	December 2022	19.6	7.0	SSE
Temporary Monitoring Station	T2-2	December 2022	20.8	5.2	NE
Temporary Monitoring Station	T3-1	December 2022	12.5	8.8	S
Temporary Monitoring Station	T3-2	December 2022	16.8	3.5	S
Temporary Monitoring Station	T3-3	December 2022	12.5	8.8	N
Temporary Monitoring Station	T4-1	December 2022	13	3.1	W
Temporary Monitoring Station	T4-2	December 2022	17.2	8.5	SSE

Note: Wind direction is recorded in 16 directional sectors (N, NNE, NE, ENE, E, ESE, SE, SSE, S, SSW, SW, WSW, W, WNW, NW, NNW), each spanning 22.5°. Due to ship drift caused by wind and ocean currents, as well as latitude and longitude measurement deviations, actual sampling positions varied somewhat between different months, resulting in fluctuations in water depth at stations with the same nominal name. For example, some stations were located at the intersection of navigation channels and shallow tidal flats, and the water depth differences during surface sediment sample collection in different months were particularly significant. Such positioning uncertainty is an inherent limitation of oceanographic operations based on ships and is considered normal. Meanwhile, based on the water depth data summarized in this table, a bathymetric contour map was constructed and is provided as Figure S1.

## Data Availability

Data are available from the corresponding authors upon reasonable request.

## Supplementary Materials

The following supporting information can be downloaded at: https://www.mdpi.com/article/10.3390/biology15151220/s1. Figure S1: Bathymetric contour map of the Southern Yellow Sea based on in situ water depth measurements at 46 stations; Figure S2: Wind rose diagram aggregated from all sampling stations in the Southern Yellow Sea during April 2020; Figure S3: Wind rose diagram aggregated from all sampling stations in the Southern Yellow Sea during May 2020; Figure S4: Wind rose diagram aggregated from all sampling stations in the Southern Yellow Sea during July 2020; Figure S5: Wind rose diagram aggregated from all sampling stations in the Southern Yellow Sea during March 2021; Figure S6: Wind rose diagram aggregated from all sampling stations in the Southern Yellow Sea during May 2021; Figure S7: Wind rose diagram aggregated from all sampling stations in the Southern Yellow Sea during November 2021; Figure S8: Wind rose diagram aggregated from all sampling stations in the Southern Yellow Sea during March 2022; Figure S9: Wind rose diagram aggregated from all sampling stations in the Southern Yellow Sea during May 2022; Figure S10: Wind rose diagram aggregated from all sampling stations in the Southern Yellow Sea during December 2022.

## References

[1] Liu Q., Cui R.F., Lin J.N., Kang Z.J., Zhou X.J. (2023). Competition relations between selected microalgae and bloom-forming *Ulva prolifera*. J. Mar. Biol. Assoc. UK.

[2] Zha M.J., Cai C.E. (2026). Identification of PHA synthesis potential and fermentation condition optimization of a *Bacillus* strain isolated from *Ulva prolifera*. Biomass Bioenergy.

[3] Zhao Y., Li Y., Jin Y., Cui T., Wang S., Kong F.L. (2022). The inhibitory effects of *Ulva prolifera* extracts on early growth of *Spartina alterniflora* and the underlying mechanisms. J. Environ. Manag..

[4] Li M., Miao X.X., Wang Y.Q., Ma X.J., Zang Y., Liu X.X., Fan S.L., Zhang X.L., Wang Z.L., Xiao J. (2025). Succession of Planktonic Crustaceans Responding to *Ulva* Green Tide in the Subei Shoal, Southwestern Yellow Sea. J. Ocean Univ. China.

[5] Senga Y., Kobayashi W., Mikawa K., Kitazawa T., Lee S.W., Shiraki Y. (2021). Influences of green macroalgae blooms on nutrients and sulfide dynamics in hypereutrophic intertidal ecosystems. Limnology.

[6] Wang C., Yu R.C., Zhou M.J. (2011). Acute toxicity of live and decomposing green alga *Ulva* (*Enteromorpha*) *prolifera* to abalone *Haliotis discus hannai*. Chin. J. Oceanol. Limnol..

[7] Sun J.C., Liu K., Zhang H.B., Fu J., Shi X.Y., Yao Z.W., Zhao G., Sha Z.X., Cui H., Wu J.P. (2025). Dissipation of *Ulva prolifera* green tides across various spatial and temporal scales and the short-term effects on marine environments. Mar. Environ. Res..

[8] Liu J.L., Xia J., Zhuang M.M., Zhang J.H., Yu K.F., Zhao S., Sun Y.Q., Tong Y.C., Xia L.H., Qin Y.T. (2021). Controlling the source of green tides in the Yellow Sea: NaClO treatment of *Ulva* attached on *Pyropia* aquaculture rafts. Aquaculture.

[9] Liu D.Y., Keesing J.K., Xing Q.G., Shi P. (2009). World’s largest macroalgal bloom caused by expansion of seaweed aquaculture in China. Mar. Pollut. Bull..

[10] Huo Y.Z., Han H.B., Shi H.H., Wu H.L., Zhang J.H., Yu K.F., Xu R., Liu C.C., Zhang Z.L., Liu K.F. (2015). Changes to the biomass and species composition of *Ulva* sp. on *Porphyra* aquaculture rafts, along the coastal radial sandbank of the Southern Yellow Sea. Mar. Pollut. Bull..

[11] Han H.B., Fan S.L., Song W., Li Y., Xiao J., Wang Z.L., Zhang X.L., Ding D.W. (2020). The contribution of attached *Ulva prolifera* on *Pyropia* aquaculture rafts to green tides in the Yellow Sea. Acta Oceanol. Sin..

[12] Huo Y.Z., Han H.B., Hua L., Wei Z.L., Yu K.F., Shi H.H., Kim J.K., Yarish C., He P.M. (2016). Tracing the origin of green macroalgal blooms based on the large scale spatio-temporal distribution of *Ulva* microscopic propagules and settled mature *Ulva* vegetative thalli in coastal regions of the Yellow Sea, China. Harmful Algae.

[13] Zhang Y.Y., He P.M., Li H.M., Li G., Liu J.H., Jiao F.L., Zhang J.H., Huo Y.Z., Shi X.Y., Su R.G. (2019). *Ulva prolifera* green-tide outbreaks and their environmental impact in the Yellow Sea, China. Natl. Sci. Rev..

[14] Keesing J.K., Liu D.Y., Fearns P., Garcia R. (2011). Inter- and intra-annual patterns of *Ulva prolifera* green tides in the Yellow Sea during 2007–2009, their origin and relationship to the expansion of coastal seaweed aquaculture in China. Mar. Pollut. Bull..

[15] Xia Z.Y., Yu J.L., Zeng Y.Q., Li M., Sun Y.Q., Tong Y.C., Liu J.L., Zhang J.H., He P.M. (2025). Continuous monitoring and comparative genetic analysis of attached outbreak species of *Ulva prolifera* in the Southern Yellow Sea. Mar. Environ. Res..

[16] Li S., Xia Z.Y., Cao J.X., Zhang J.H., He P.M. (2024). Distribution of *Ulva* macroalgae before and after the outbreak of the Yellow Sea green tide in aquaculture ponds and rivers nearshore Jiangsu, China. Reg. Stud. Mar. Sci..

[17] Xia Z.Y., Yuan H.Q., Liu J.L., Zhao S., Tong Y.C., Sun Y.Q., Li S., Li A.Q., Cao J.X., Xia J. (2022). Biomass and species composition of green macroalgae in the Binhai Harbor intertidal zone of the southern Yellow Sea. Mar. Pollut. Bull..

[18] Xia Z.Y., Liu J.L., Zhao S., Cui Q.W., Bi F.L., Zhang J.H., He P.M. (2023). Attached *Ulva meridionalis* on nearshore dikes may pose a new ecological risk in the Yellow Sea. Environ. Pollut..

[19] Pang S.J., Liu F., Shan T.F., Xu N., Zhang Z.H., Gao S.Q., Chopin T., Sun S. (2010). Tracking the algal origin of the *Ulva* bloom in the Yellow Sea by a combination of molecular, morphological and physiological analyses. Mar. Environ. Res..

[20] Liu S.H., Liu C.C., Xu R., Qin Y.T., Deng B.P., Ji X., Yuan Y.M., Xia L.H., He P.M. (2016). Analysis of relationships between onshore aquaculture ponds in Jiangsu and the source of green tide in the South Yellow Sea. Mar. Fish..

[21] Sun Y.Q., Yao L.L., Liu J.L., Tong Y.C., Xia J., Zhao X.H., Zhao S., Fu M.L., Zhuang M.M., He P.M. (2022). Prevention strategies for green tides at source in the Southern Yellow Sea. Mar. Pollut. Bull..

[22] Huan L., Shi M.M., Wang X.L., Gu W.H., Zhang B.Y., Liu X.H., Zhuo J.T., Wang G.C. (2023). Morphological characteristics and genetic diversity of floating and attached *Ulva prolifera*––A case study in the Yellow Sea, China. Mar. Pollut. Bull..

[23] Li D.X., Gao Z.Q., Song D.B. (2021). Analysis of environmental factors affecting the large-scale long-term sequence of green tide outbreaks in the Yellow Sea. Estuar. Coast. Shelf Sci..

[24] Li D.X., Gao Z.Q., Song D.B. (2022). Analysis of the reasons for the outbreak of Yellow Sea green tide in 2021 based on long-term multi-source data. Mar. Environ. Res..

[25] Li Y.F., Geng H.X., Hong X., Kong F.Z., Yu R.C. (2025). Strong interannual variation of green tides in the southern Yellow Sea: Crucial factors and implications on management strategies. Mar. Pollut. Bull..

[26] Xia Z.Y., Liu J.L., Zhao S., Sun Y.Q., Cui Q.W., Wu L.J., Gao S., Zhang J.H., He P.M. (2024). Review of the development of the green tide and the process of control in the southern Yellow Sea in 2022. Estuar. Coast. Shelf Sci..

[27] Xia L.H., Qin Y.T., Ji H.H., Cao J.X., Wang X.B., Zhang Y.H., Liu J.L. (2026). Spatiotemporal Dynamics of Micropropagules in Seawater During the 2020 Green Tide Outbreak in the Southern Yellow Sea. Biology.

[28] CCTV.com Experts Discuss *Ulva* Control: Land-Sea Coordination Is Key to Reducing Marine Eutrophication. 2021. (In Chinese). https://ocean.cctv.com/2021/07/27/ARTIV2mdIMDxj7fVuq7trXYn210714.shtml.

[29] Yao W.M., Lei Y.Y., Tan S.L., Qin Y.T., Ji H.H., Sun Y.Q., Zhang J.H., Liu J.L. (2025). Distribution Characteristics of Suspended Macroalgae in the Southern Yellow Sea Before the Green Tide Outbreak. Biology.

[30] Cao J.X., Liu J.L., Zhao S., Tong Y.C., Li S., Xia Z.Y., Hu M.J., Sun Y.Q., Zhang J.H., He P.M. (2023). Advances in the research on micropropagules and their role in green tide outbreaks in the Southern Yellow Sea. Mar. Pollut. Bull..

[31] Li A.Q., Zhao S., Sun J.Y., Liu H.T., Sun Y.Q., Bi F.L., Xia Z.Y., Dai W., He W.H., Zhang J.H. (2024). Overwintering and summer survival of *Ulva prolifera* in sediments: Indoor simulation of temperature impacts. Mar. Pollut. Bull..

[32] Yu F., Ren Q., Diao X.Y., Wei C.J., Hu Y.B. (2022). The Sandwich Structure of the Southern Yellow Sea Cold Water Mass and Yellow Sea Warm Current. Front. Mar. Sci..

[33] Wang Y., Liu Y.J., Guo H., Zhang H.B., Li D.M., Yao Z.W., Wang X.C., Jia C. (2022). Long-term nutrient variation trends and their potential impact on phytoplankton in the southern Yellow Sea, China. Acta Oceanol. Sin..

[34] Xu Y.N., Xu T.B. (2022). An evolving marine environment and its driving forces of algal blooms in the Southern Yellow Sea of China. Mar. Environ. Res..

[35] Song M.J., Yan T., Kong F.Z., Wang Y.F., Zhou M.J. (2022). Increased diversity and environmental threat of harmful algal blooms in the Southern Yellow Sea, China. J. Oceanol. Limnol..

[36] Wu S.G., Ni X.N., Cai F. (2008). Petroleum geological framework and hydrocarbon potential in the Yellow Sea. Chin. J. Oceanol. Limnol..

[37] Han H.B., Song W., He P.M., Wang Z.L., Ding D.W. (2018). Tempo-spatial distribution of green algae micro-propagules in the Yellow Sea after the outbreak of the green tide in 2014. Mar. Environ. Sci..

[38] Liu X.Q., Wang Z.L., Zhang X.L., Li R.X., Li Y., Fan S.L., Song W. (2015). The Current Situations of Micro-Propagules of Green Algae in *Porphyra yezoensis* Farming Areas of Southern Jiangsu in Autumn in 2012. Period. Ocean Univ. China.

[39] Huo Y.Z., Zhang J.H., Chen L.P., Hu M., Yu K.F., Chen Q.F., He Q., He P.M. (2013). Green algae blooms caused by *Ulva prolifera* in the Southern Yellow Sea: Identification of the original bloom location and evaluation of biological processes occurring during the early northward floating period. Limnol. Oceanogr..

[40] Ding L.P., Luan R.X. (2013). Flora Algarum Marinarum Sinicarum. Tomus IV, Chlorophyta. No.I, Ulotrichales Chaetophorales Phaeophilales Ulvales Prasiolales Cladophorales Acrosiphoniales.

[41] Han W., Chen L.P., Zhang J.H., Tian X.L., Hua L., He Q., Huo Y.Z., Yu K.F., Shi D.J., Ma J.H. (2013). Seasonal variation of dominant free-floating and attached *Ulva* species in Rudong coastal area, China. Harmful Algae.

[42] Wang S.Y., Huo Y.Z., Zhang J.H., Cui J.J., Wang Y., Yang L.L., Zhou Q.Y., Lu Y.W., Yu K.F., He P.M. (2018). Variations of dominant free-floating *Ulva* species in the source area for the world’s largest macroalgal blooms, China: Differences of ecological tolerance. Harmful Algae.

[43] Miao X.X., Xiao J., Pang M., Zhang X.L., Wang Z.L., Miao J.W., Li Y. (2018). Effect of the large-scale green tide on the species succession of green macroalgal micro-propagules in the coastal waters of Qingdao, China. Mar. Pollut. Bull..

[44] Song W., Li Y., Fang S., Wang Z.L., Xiao J., Li R.X., Fu M.Z., Zhu M.Y., Zhang X.L. (2015). Temporal and spatial distributions of green algae micro-propagules in the coastal waters of the Subei Shoal, China. Estuar. Coast. Shelf Sci..

[45] Ma X.J., Miao X.X., Fan S.L., Zang Y., Zhang B.T., Li M., Zhang X.L., Fu M.Z., Wang Z.L., Xiao J. (2024). Dynamics of green macroalgal micro-propagules and the influencing factors in the southern Yellow Sea, China. Sci. Total Environ..

[46] Cui J.J., Shi J.T., Zhang J.H., Wang L.T., Fan S.Y., Xu Z.Y., Huo Y.Z., Zhou Q.Y., Lu Y.W., He P.M. (2018). Rapid expansion of *Ulva* blooms in the Yellow Sea, China through sexual reproduction and vegetative growth. Mar. Pollut. Bull..

[47] Shao K.S., Gong N., Shen L.Y., Han X., Wang Z.X., Zhou K., Kong D.Y., Pan X.S., Cong P.F. (2024). Why did the world’s largest green tides occur exclusively in the southern Yellow Sea?. Mar. Environ. Res..

[48] Li Y., Song W., Xiao J., Wang Z.L., Fu M.Z., Zhu M.Y., Li R.X., Zhang X.L., Wang X.N. (2014). Tempo-spatial distribution and species diversity of green algae micro-propagules in the Yellow Sea during the large-scale green tide development. Harmful Algae.

[49] Zhao J., Kong F.Z., Liu Q.C., Li F.J., Wei X., Yan T., Jiang P. (2022). Tempo-spatial distribution of *Ulva* spp. micro-propagules in the Yellow Sea during and after green tide in 2019. J. Oceanol. Limnol..

[50] Ren C.G., Zhong Z.H., Liu Z.Y., Lin S., Luo Y.K., Qin S. (2024). The ever-lasting green tides: What can we do?. Heliyon.

[51] Xiao J., Wang Z.L., Liu D.Y., Fu M.Z., Yuan C., Yan T. (2021). Harmful macroalgal blooms (HMBs) in China’s coastal water: Green and golden tides. Harmful Algae.

[52] Feng Y., Xiong Y.L., Hall-Spencer J.M., Liu K.L., Beardall J., Gao K.S., Ge J.K., Xu J.T., Gao G. (2024). Shift in algal blooms from micro- to macroalgae around China with increasing eutrophication and climate change. Glob. Change Biol..

[53] Xiao M.Y., Song W.N., Zhang H.B., Shi X.Y., Su R.G. (2024). Eutrophication of Jiangsu Coastal Water and Its Role in the Formation of Green Tide. J. Ocean Univ. China.

[54] Zhang H.B., Su R.G., Shi X.Y., Zhang C.S., Yin H., Zhou Y.L., Wang G.S. (2020). Role of nutrients in the development of floating green tides in the Southern Yellow Sea, China, in 2017. Mar. Pollut. Bull..

[55] Liu X.Q., Wang Z.L., Zhang X.L. (2016). A review of the green tides in the Yellow Sea, China. Mar. Environ. Res..

[56] Wang Z.L., Xiao J., Yuan C., Miao X.X., Fan S.L., Fu M.Z., Xia T., Zhang X.L. (2023). The drifting and spreading mechanism of floating *Ulva* mass in the waterways of Subei shoal, the Yellow Sea of China-Application for abating the world’s largest green tides. Mar. Pollut. Bull..

[57] Jiang X.P., Gao Z.Q., Zhang Q.C., Wang Y.Q., Tian X.P., Shang W.T., Xu F.X. (2020). Remote sensing methods for biomass estimation of green algae attached to nursery-nets and raft rope. Mar. Pollut. Bull..

[58] Xu F.X., Gao Z.Q., Jiang X.P., Shang W.T., Ning J.C., Song D.B., Ai J.Q. (2018). A UAV and S2A data-based estimation of the initial biomass of green algae in the South Yellow Sea. Mar. Pollut. Bull..

[59] Moulager M., Corellou F., Vergé V., Escande M.L., Bouget F.Y. (2010). Integration of Light Signals by the Retinoblastoma Pathway in the Control of S Phase Entry in the Picophytoplanktonic Cell *Ostreococcus*. PLoS Genet..

[60] Wagner V., Gessner G., Mittag M. (2005). Functional proteomics: A promising approach to find novel components of the circadian system. Chronobiol. Int..

[61] Kalita T.L., Titlyanova T.V., Titlyanov E.A. (2007). New rhythmic changes in mitosis and growth in low differentiated green and red marine macroalgae. Russ. J. Mar. Biol..

[62] Schmid R., Forster R., Dring M.J. (1992). Circadian-rhythm and fast responses to blue-light of photosynthesis in ectocarpus (Phaeophyta, Ectocarpales). 2. Light and CO_2_ dependence of photosynthesis. Planta.

[63] Schmid R., Dring M.J. (1992). Circadian-rhythm and fast responses to blue-light of photosynthesis in ectocarpus (Phaeophyta, Ectocarpales). 1. Characterization of the rhythm and the blue-light response. Planta.

[64] Schmid R., Dring M.J., Forster R.M. (1994). Kinetics of blue-light stimulation and circadian rhythmicity of light-saturated photosynthesis in brown-algae—A species comparison. J. Phycol..

[65] Dearaujo F.F.T., Pires M.A., Frankel R.B., Bicudo C.E.M. (1986). Magnetite and Magnetotaxis in Bacteria and Algae. Biophys. J..

[66] Salta M., Wharton J.A., Blache Y., Stokes K.R., Briand J.F. (2013). Marine biofilms on artificial surfaces: Structure and dynamics. Environ. Microbiol..

[67] Petroff A.P., Hernandez J., Kelin V., Radchenko-Hannafin N. (2025). Magnetotactic bacteria optimally navigate natural pore networks. eLife.

[68] Rahul P.S., Priya Y., Himani S., Rajan K.G. (2024). Sustainable approaches in microalgal biomass harvesting: Current advances and future perspectives. Int. J. Agric. Biosci..

[69] Xia Z.Y., Cao X.L., Li S., Cao J.X., Tong Y.C., Sun Y.Q., Liu J.L., Zhao S., Cui Q.W., Zeng Y.Q. (2023). Distribution of *Ulva prolifera*, the dominant species in green tides along the Jiangsu Province coast in the Southern Yellow Sea, China. J. Sea Res..

[70] Zhao J., Jiang P., Liu Z.Y., Wei W., Lin H.Z., Li F.C., Wang J.F., Qin S. (2013). The yellow sea green tides were dominated by one species, *Ulva* (*Enteromorpha*) *prolifera*, from 2007 to 2011. Chin. Sci. Bull..

[71] Li Y., Xiao J., Ding L.P., Wang Z.L., Song W., Fang S., Fan S.L., Li R.X., Zhang X.L. (2015). Community structure and controlled factor of attached green algae on the *Porphyra yezoensis* aquaculture rafts in the Subei Shoal, China. Acta Oceanol. Sin..

[72] Song W., Jiang M.J., Wang Z.L., Wang H.P., Zhang X.L., Fu M.Z. (2018). Source of propagules of the fouling green macroalgae in the Subei Shoal, China. Acta Oceanol. Sin..

[73] Chen L.H., Cao X.B., Xing R.L., Jiang A.L. (2020). Effects of *Chaetomorpha valida* bloom development on the structure and succession of the phytoplankton community in *Apostichopus japonicus* culture ponds. Aquac. Res..

[74] Lin G.R., Sun F.L., Wang C.Z., Zhang L., Zhang X.Z. (2017). Assessment of the effect of *Enteromorpha prolifera* on bacterial community structures in aquaculture environment. PLoS ONE.

[75] Shin H.W. (2008). Rapid attachment of spores of the fouling alga *Ulva fasciata* on biofilms. J. Environ. Biol..

[76] Geng H.X., Yan T., Zhou M.J., Liu Q. (2015). Comparative study of the germination of *Ulva prolifera* gametes on various substrates. Estuar. Coast. Shelf Sci..

[77] Park C.S., Hwang E.K. (2011). An investigation of the relationship between sediment particles size and the development of green algal mats (*Ulva prolifera*) on the intertidal flats of Muan, Korea. J. Appl. Phycol..

[78] Park S.R., Kang Y.H., Lee H.J., Ko Y.W., Kim J.H. (2014). The importance of substratum and elevation in recruitment and persistence of ulvoid algal blooms on rocky intertidal shores of the southern Korean coast. Bot. Mar..

[79] Thirumurugan N.K., Dhinakarasamy I., Chakraborty S., Sivakumar M., Clements C., Chandrasekar A., Vinayagam J., Kumar C., Rajendran T. (2024). Synergistic effects of plastic debris and elevated nitrate concentrations on the proliferation of *Ulva lactuca* micro-propagules. J. Hazard. Mater..

[80] Liu F., Pang S.J., Chopin T., Gao S.Q., Shan T.F., Zhao X.B., Li J. (2013). Understanding the recurrent large-scale green tide in the Yellow Sea: Temporal and spatial correlations between multiple geographical, aquacultural and biological factors. Mar. Environ. Res..

[81] Shen Q., Li H.Y., Li Y., Wang Z.L., Liu J.S., Yang W.D. (2012). Molecular identification of green algae from the rafts based infrastructure of *Porphyra yezoensis*. Mar. Pollut. Bull..

[82] Zhang L., Wang G.L., Liu C., Chi S., Liu T. (2014). Development and utility of EST-SSR markers in *Ulva prolifera* of the South Yellow Sea. Acta Oceanol. Sin..

[83] Han H.B., Li Y., Ma X.J., Song W., Wang Z.L., Fu M.Z., Zhang X.L. (2022). Population differentiation in the dominant species (*Ulva prolifera*) of green tide in coastal waters of China. Acta Oceanol. Sin..

[84] Sun S., Ma W.W., Wang N., Feng S., Sun Y. (2024). Genome-wide SNP markers provided insights into the reproductive strategy and genetic diversity of the green tide causative species *Ulva prolifera* in China. J. Oceanol. Limnol..

[85] Zheng X.Y., Li J.H., Hou Y.Z., Jiang S.S., Xing Q.G. (2025). Estimating windage coefficient of floating marine green tide of *Ulva prolifera* using UAV optical images. Mar. Pollut. Bull..

[86] Wang S., Zhao L., Wang Y.H., Zhang H.Y., Li F., Zhang Y.J. (2022). Distribution characteristics of green tides and its impact on environment in the Yellow Sea. Mar. Environ. Res..

[87] Zhao S., Liu J.L., Xia Z.Y., Sun J.Y., Zhang J.H., He P.M. (2025). Subtypes I and II of *Ulva prolifera* of Müller: Dominant Green Tide Species in the Southern Yellow Sea and Their Responses to Natural Light and Temperature Conditions. Biology.

[88] Jahan K., Kristyanto S., Choi K.H. (2025). Genome-Wide DNA Methylation and Transcription Analysis Reveal the Potential Epigenetic Mechanism of Heat-Light Stress Response in the Green Macro Algae *Ulva prolifera*. Int. J. Mol. Sci..

[89] Cui J.J., Zhang J.H., Huo Y.Z., Zhou L.J., Wu Q., Chen L.P., Yu K.F., He P.M. (2015). Adaptability of free-floating green tide algae in the Yellow Sea to variable temperature and light intensity. Mar. Pollut. Bull..

[90] Zhao Z.F., Liu Z.Y., Qin S., Wang X.H., Song W.L., Liu K., Zhuang L.C., Xiao S.Z., Zhong Z.H. (2021). Impacts of low pH and low salinity induced by acid rain on the photosynthetic activity of green tidal alga *Ulva prolifera*. Photosynthetica.

[91] Wu H.L., Liu Y.M., Beardall J., Zhong Z.H., Gao G., Xu J.T. (2022). Physiological acclimation of *Ulva prolifera* to seasonal environmental factors drives green tides in the Yellow Sea. Mar. Environ. Res..

[92] Gao G., Zhong Z.H., Zhou X.H., Xu J.T. (2016). Changes in morphological plasticity of *Ulva prolifera* under different environmental conditions: A laboratory experiment. Harmful Algae.

[93] Yamaha K., Nishihara G.N., Sato Y., Inomata E. (2025). Interactive effects of CO_2_ concentration and temperature on photosynthesis and growth of cultivated *Ulva prolifera* germling clusters. J. Appl. Phycol..

[94] Han H.B., Zhao S., Song X.L., Wang H. (2023). The Overwintering Capability of *Ulva prolifera* Spores and Gametes in the Yellow Sea, China. J. Ocean Univ. China.

[95] Song W., Peng K.Q., Xiao J., Li Y., Wang Z.L., Liu X.Q., Fu M.Z., Fan S.L., Zhu M.Y., Li R.X. (2015). Effects of temperature on the germination of green algae micro-propagules in coastal waters of the Subei Shoal, China. Estuar. Coast. Shelf Sci..

[96] Jiang J.A., Chen Y.L., Zhang R.H., Zhu W.R., Liu F.J., Xu N.J., Li Y.H. (2025). New insights on the impact of light, photoperiod and temperature on the reproduction of green algae *Ulva prolifera* via transcriptomics and physiological analyses. Mar. Pollut. Bull..

[97] Chen B.N., Zou D.H. (2015). Altered seawater salinity levels affected growth and photosynthesis of *Ulva fasciata* (Ulvales, Chlorophyta) germlings. Acta Oceanol. Sin..

[98] Cardoso I., Meissner A., Sawicki A., Bartsch I., Valentin K.U., Steinhagen S., Buck B.H., Hofmann L.C. (2023). Salinity as a tool for strain selection in recirculating land-based production of *Ulva* spp. from germlings to adults. J. Appl. Phycol..

[99] Mantri V.A., Singh R.P., Bijo A.J., Kumari P., Reddy C.R.K., Jha B. (2011). Differential response of varying salinity and temperature on zoospore induction, regeneration and daily growth rate in *Ulva fasciata* (Chlorophyta, Ulvales). J. Appl. Phycol..

[100] Imchen T. (2012). Recruitment Potential of a Green Alga *Ulva flexuosa* Wulfen Dark Preserved Zoospore and Its Development. PLoS ONE.

[101] Liu J.L., Xia Z.Y., Zeng Y.Q., Xia J., He P.M. (2025). Exploration and implication of green macroalgal proliferation in the Nanhui-east-tidal-flat: An investigation of post-reclamation mudflat wetlands. Front. Mar. Sci..

[102] Xia Z.Y., Yang Y.T., Zeng Y.Q., Sun Y.Q., Cui Q.W., Chen Z.H., Liu J.L., Zhang J.H., He P.M. (2024). Temporal succession of micropropagules during accumulation and dissipation of green tide algae: A case study in Rudong coast, Jiangsu Province. Mar. Environ. Res..

[103] Rossi F. (2006). Small-scale burial of macroalgal detritus in marine sediments: Effects of *Ulva* spp. on the spatial distribution of macrofauna assemblages. J. Exp. Mar. Biol. Ecol..

[104] Karlson A.M.L., Pilditch C.A., Probert P.K., Leduc D., Savage C. (2021). Large Infaunal Bivalves Determine Community Uptake of Macroalgal Detritus and Food Web Pathways. Ecosystems.

[105] Chen J., Li H.M., Zhang Z.H., He C., Shi Q., Jiao N.Z., Zhang Y.Y. (2020). DOC dynamics and bacterial community succession during long-term degradation of *Ulva prolifera* and their implications for the legacy effect of green tides on refractory DOC pool in seawater. Water Res..

[106] Castaldelli G., Welsh D.T., Flachi G., Zucchini G., Colombo G., Rossi R., Fano E.A. (2003). Decomposition dynamics of the bloom forming macroalga *Ulva rigida* C. Agardh determined using a ^14^C-carbon radio-tracer technique. Aquat. Bot..

[107] Liu X.X., Zang Y., Fan S.L., Miao X.X., Fu M.Z., Ma X.J., Li M., Zhang X.L., Wang Z.L., Xiao J. (2024). Changes in the structure of the microbial community within the phycospheric microenvironment and potential biogeochemical effects induced in the demise stage of green tides caused by *Ulva prolifera*. Front. Microbiol..

[108] Li H.M., Feng X.T., Xiong T.Q., Zhang Z.H., Huang S.R., Zhang Y.Y. (2025). Herbivore grazing enhances macroalgal organic carbon release and alters their carbon sequestration fate in the ocean. Mar. Environ. Res..

[109] Martinetto P., Teichberg M., Valiela I., Montemayor D., Iribarne O. (2011). Top-down and bottom-up regulation in a high nutrient-high herbivory coastal ecosystem. Mar. Ecol. Prog. Ser..

[110] Huo Y.Z., Kim J.K., Yarish C., Augyte S., He P.M. (2021). Responses of the germination and growth of *Ulva prolifera* parthenogametes, the causative species of green tides, to gradients of temperature and light. Aquat. Bot..

[111] Bender V.B., Hanebuth T.J.J., Mena A., Baumann K.H., Francés G., von Dobeneck T. (2012). Control of sediment supply, palaeoceanography and morphology on late Quaternary sediment dynamics at the Galician continental slope. Geo-Mar. Lett..

[112] Mogg A.O.M., Attard K.M., Stahl H., Brand T., Turnewitsch R., Sayer M.D.J. (2017). The influence of coring method on the preservation of sedimentary and biogeochemical features when sampling soft-bottom, shallow coastal environments. Limnol. Oceanogr. Methods.

[113] Crusius J., Anderson R.F. (1991). Core compression and surficial sediment loss of lake-sediments of high porosity caused by gravity coring. Limnol. Oceanogr..

[114] Foucher A., Chaboche P.A., Sabatier P., Evrard O. (2021). A worldwide meta-analysis (1977–2020) of sediment core dating using fallout radionuclides including ^137^Cs and ^210^Pb_xs_. Earth Syst. Sci. Data.

[115] Li W.P., Li X.X., Mei X., Zhang F., Xu J.P., Liu C.R., Wei C.A.Y., Liu Q.S. (2021). A review of current and emerging approaches for Quaternary marine sediment dating. Sci. Total Environ..

[116] Liu F., Liu X.F., Jin Z., Yu R.C., Kong F.Z., Yan T. (2018). Vertical distribution and species diversity of microscopic propagules of green macroalgae in sediments of Subei Shoal, China. Oceanol. Limnol. Sin..

