# Supplementary material for: Spatiotemporal Distribution of Ulva Micropropagules in Surface Sediments: Insights from Field Sampling Data in the Southern Yellow Sea (2020–2022)
Source: Biology (Basel). 2026 Jul 23;15(15):1220. doi: 10.3390/biology15151220 (PMC13465260; doi:10.3390/biology15151220)
Supplement: Supplementary file 1 [file biology-15-01220-s001.zip › biology-4422070-supplementary.pdf]

SUPPLEMENTAL INFORMATION

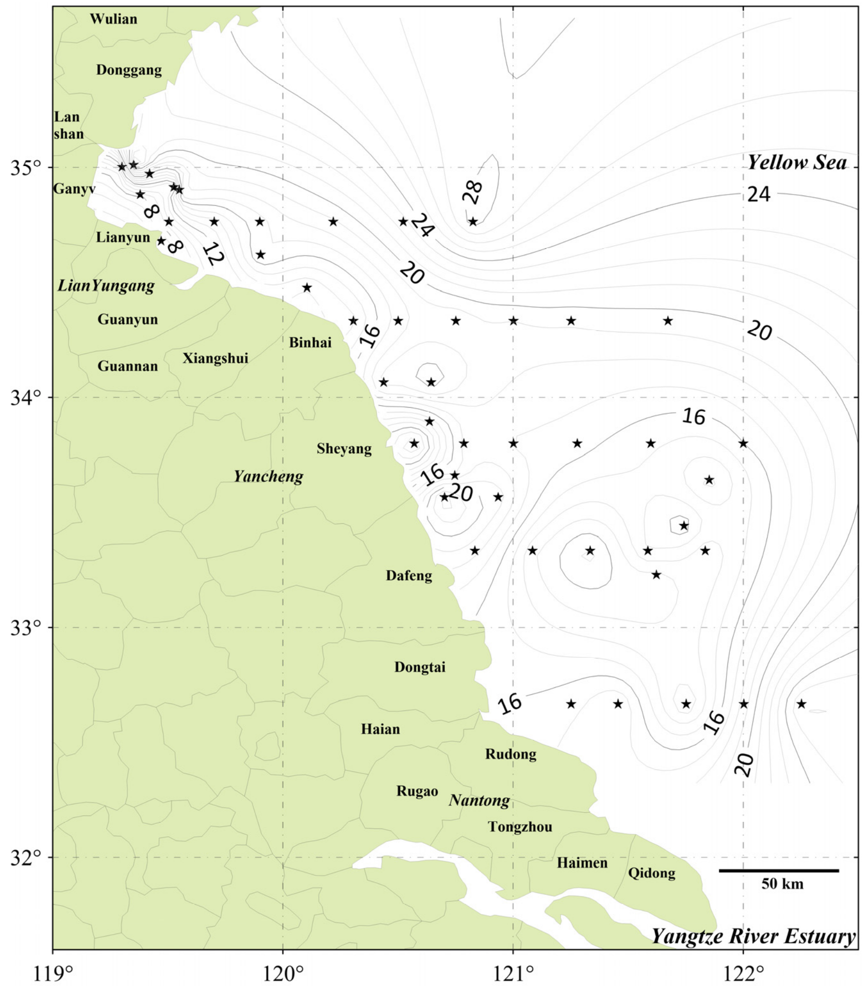

Figure S1. Bathymetric contour map of the Southern Yellow Sea based on in situ water depth measurements at 46 stations.

Note: Black stars represent stations; specific station numbers can be obtained from Figure 1. In Figure S1, solid lines represent isobaths, and the numbers alongside them indicate seawater depths calculated using the Kriging interpolation algorithm in Surfer 9 software, with units in meters (m). Ocean depth often exhibits marked regional variations due to factors such as topographic relief, tidal fluctuations, and differences in substrate types. The bathymetric contour lines presented in this figure were extrapolated from in situ water depth measurements collected at 46 stations in the Southern Yellow Sea. They reflect the general trend and variation pattern of water depth distribution at the macroscopic scale. However, constrained by the limited number of stations and spatial coverage, the interpolation results serve only as indicative trends and references, and cannot accurately resolve the fine-scale bathymetric details of local micro-topography. Therefore, the isobath distribution and annotated depth values shown in this figure are for reference only. In actual research and application, the water depth data for specific regions should ultimately be based on field survey measurements obtained from in situ marine investigations.

The original water depth data used for contour mapping are detailed in Table 1. The following

processing protocol was applied to the depth measurements at each station: when the number of depth determinations at a station equaled one, that single measured value was adopted directly as the water depth for that station; when the number of determinations was two or more, the arithmetic mean of the multiple measurements was taken as the water depth for that station. Subsequently, the mean water depth of each station was used as the fundamental dataset for spatial interpolation, imported into Surfer 9 software, and the Kriging interpolation algorithm was selected for spatial analysis and computation to produce a continuous bathymetric distribution.

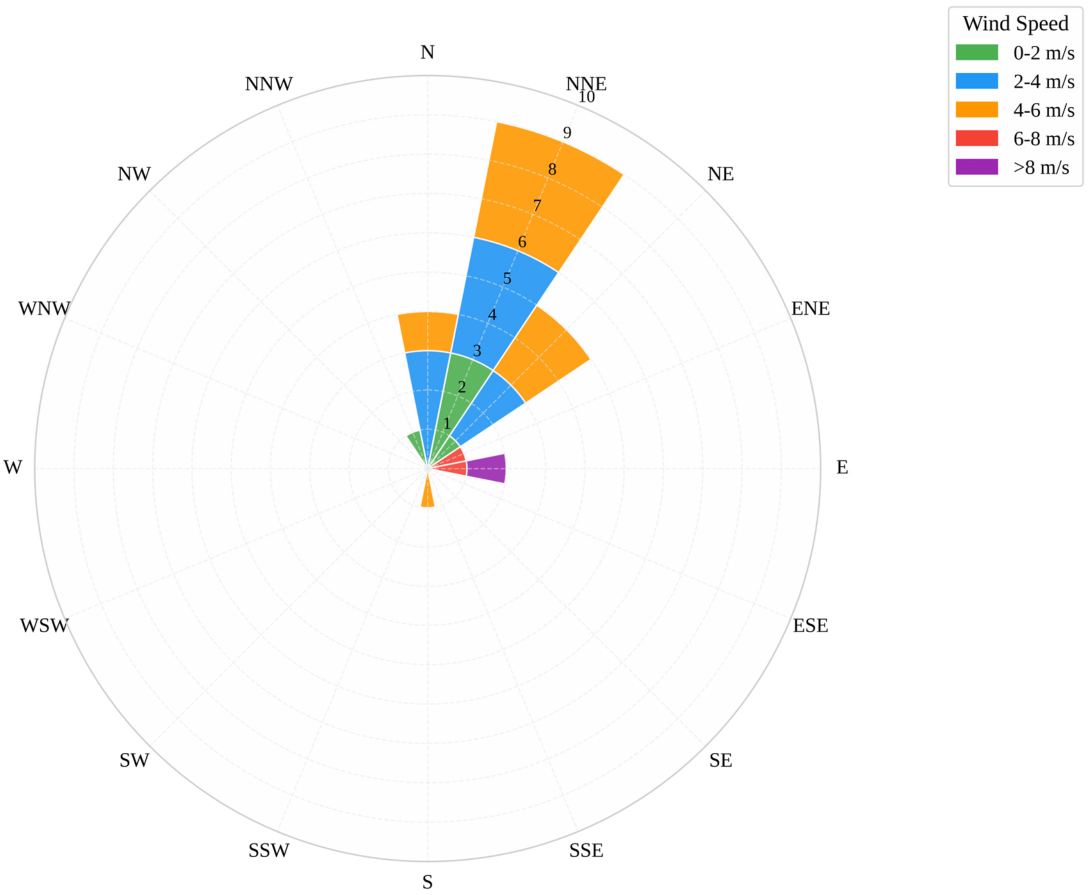

Figure S2. Wind rose diagram aggregated from all sampling stations in the Southern Yellow Sea during April 2020.

Note: Wind speed was categorized into five classes: [0, 2], (2, 4], (4, 6], (6, 8], and >8 m/s, with detailed information provided in Table 1. The data presented in this figure were obtained from in situ measurements of wind direction and wind speed at 23 stations in the Southern Yellow Sea during the cruise survey of the corresponding month. As the sampling period covered only selected days within the month, the limited spatial and temporal coverage means the results may not fully reflect the complete wind field characteristics for that month. It should be noted that the Southern Yellow Sea is extensive, and the pronounced thermal contrast between land and sea leads to significant diurnal variations in wind direction; therefore, the aggregated wind rose diagram cannot fully represent the local wind field characteristics at individual stations. For more detailed raw data, including daily mean wind speed, maximum wind speed, and corresponding wind direction at each station, users are advised to access the public database of the National Oceanic and Atmospheric Administration (NOAA) at https://www.ncei.noaa.gov/ to download observational records for the relevant period.

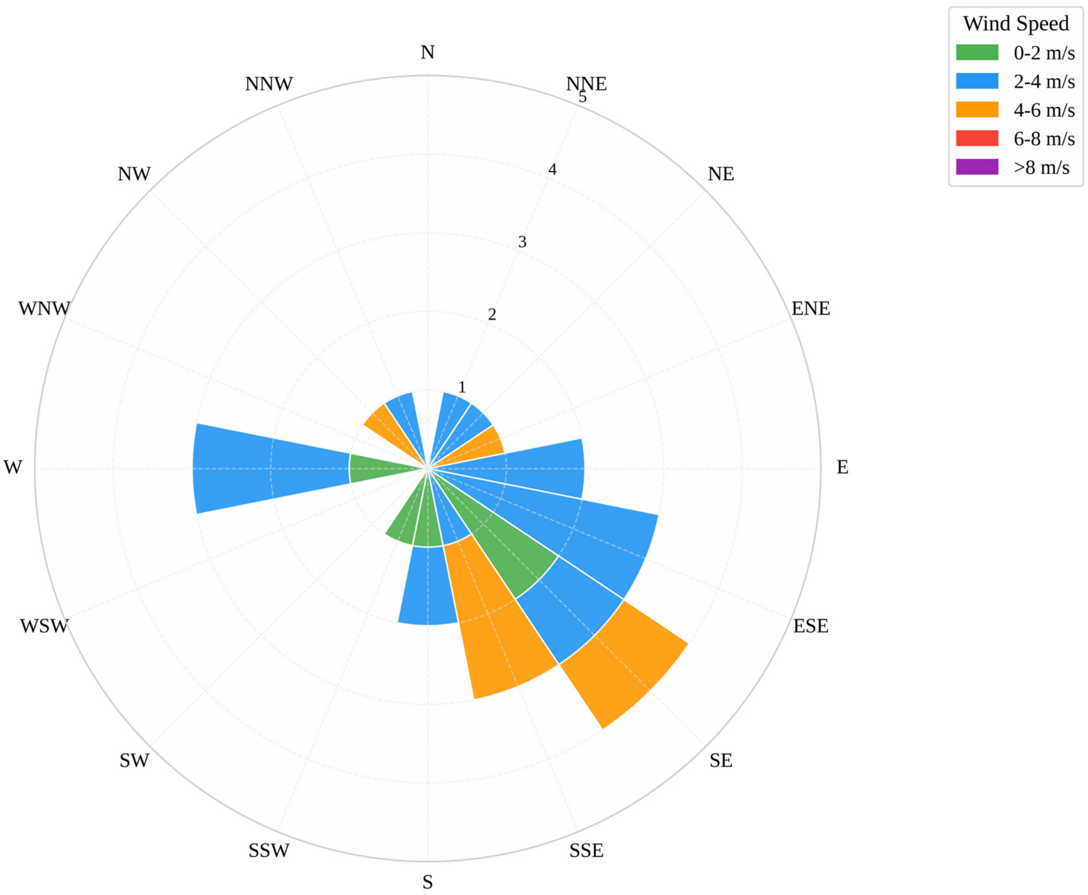

Figure S3. Wind rose diagram aggregated from all sampling stations in the Southern Yellow Sea during May 2020.

Note: Wind speed was categorized into five classes: [0, 2], (2, 4], (4, 6], (6, 8], and >8 m/s, with detailed information provided in Table 1. The data presented in this figure were obtained from in situ measurements of wind direction and wind speed at 23 stations in the Southern Yellow Sea during the cruise survey of the corresponding month. As the sampling period covered only selected days within the month, the limited spatial and temporal coverage means the results may not fully reflect the complete wind field characteristics for that month. It should be noted that the Southern Yellow Sea is extensive, and the pronounced thermal contrast between land and sea leads to significant diurnal variations in wind direction; therefore, the aggregated wind rose diagram cannot fully represent the local wind field characteristics at individual stations. For more detailed raw data, including daily mean wind speed, maximum wind speed, and corresponding wind direction at each station, users are advised to access the public database of the National Oceanic and Atmospheric Administration (NOAA) at https://www.ncei.noaa.gov/ to download observational records for the relevant period.

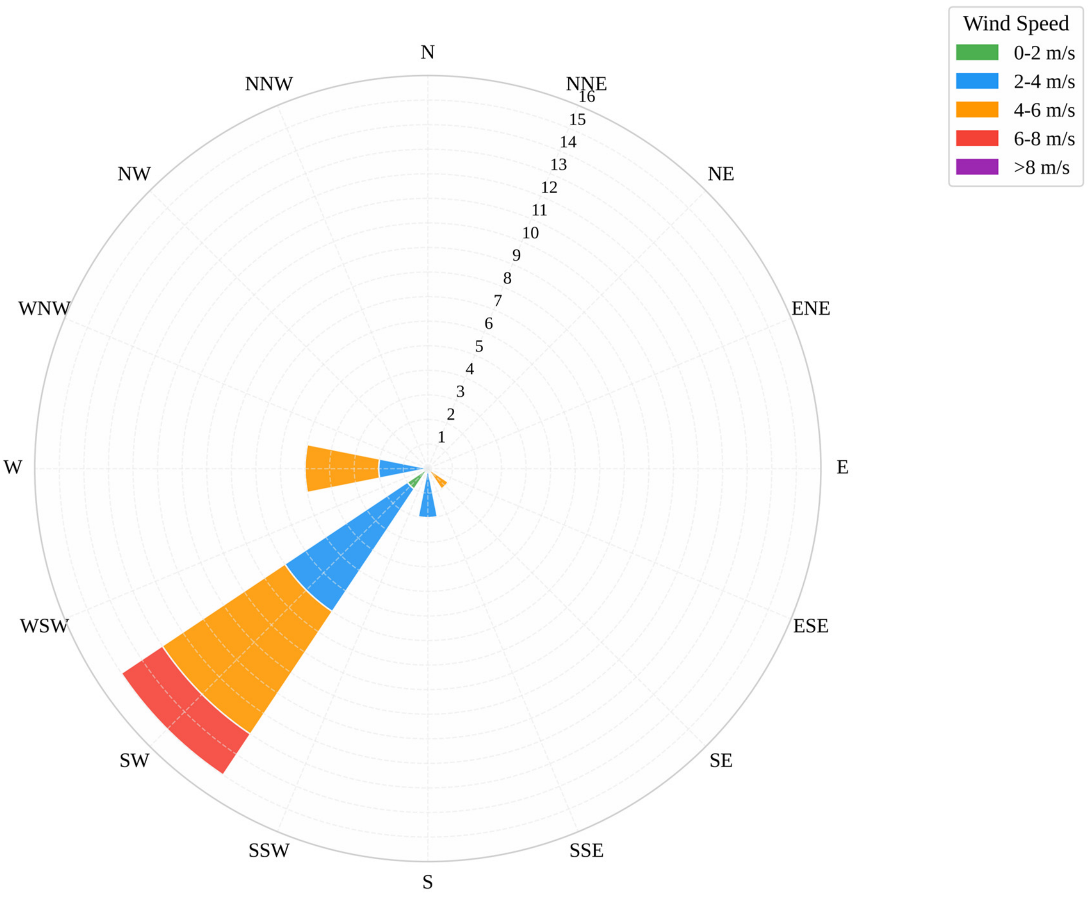

Figure S4. Wind rose diagram aggregated from all sampling stations in the Southern Yellow Sea during July 2020.

Note: Wind speed was categorized into five classes: [0, 2], (2, 4], (4, 6], (6, 8], and >8 m/s, with detailed information provided in Table 1. The data presented in this figure were obtained from in situ measurements of wind direction and wind speed at 23 stations in the Southern Yellow Sea during the cruise survey of the corresponding month. As the sampling period covered only selected days within the month, the limited spatial and temporal coverage means the results may not fully reflect the complete wind field characteristics for that month. It should be noted that the Southern Yellow Sea is extensive, and the pronounced thermal contrast between land and sea leads to significant diurnal variations in wind direction; therefore, the aggregated wind rose diagram cannot fully represent the local wind field characteristics at individual stations. For more detailed raw data, including daily mean wind speed, maximum wind speed, and corresponding wind direction at each station, users are advised to access the public database of the National Oceanic and Atmospheric Administration (NOAA) at https://www.ncei.noaa.gov/ to download observational records for the relevant period.

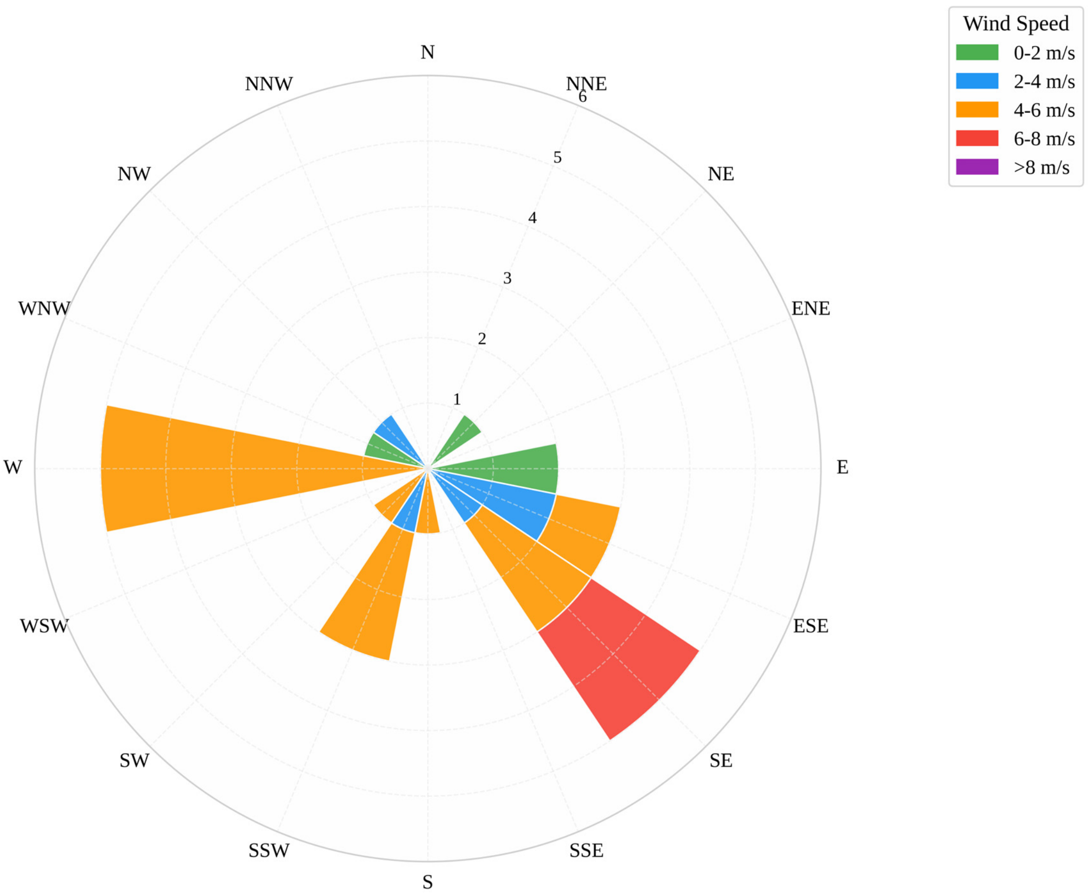

Figure S5. Wind rose diagram aggregated from all sampling stations in the Southern Yellow Sea during March 2021.

Note: Wind speed was categorized into five classes: [0, 2], (2, 4], (4, 6], (6, 8], and >8 m/s, with detailed information provided in Table 1. The data presented in this figure were obtained from in situ measurements of wind direction and wind speed at 23 stations in the Southern Yellow Sea during the cruise survey of the corresponding month. As the sampling period covered only selected days within the month, the limited spatial and temporal coverage means the results may not fully reflect the complete wind field characteristics for that month. It should be noted that the Southern Yellow Sea is extensive, and the pronounced thermal contrast between land and sea leads to significant diurnal variations in wind direction; therefore, the aggregated wind rose diagram cannot fully represent the local wind field characteristics at individual stations. For more detailed raw data, including daily mean wind speed, maximum wind speed, and corresponding wind direction at each station, users are advised to access the public database of the National Oceanic and Atmospheric Administration (NOAA) at https://www.ncei.noaa.gov/ to download observational records for the relevant period.

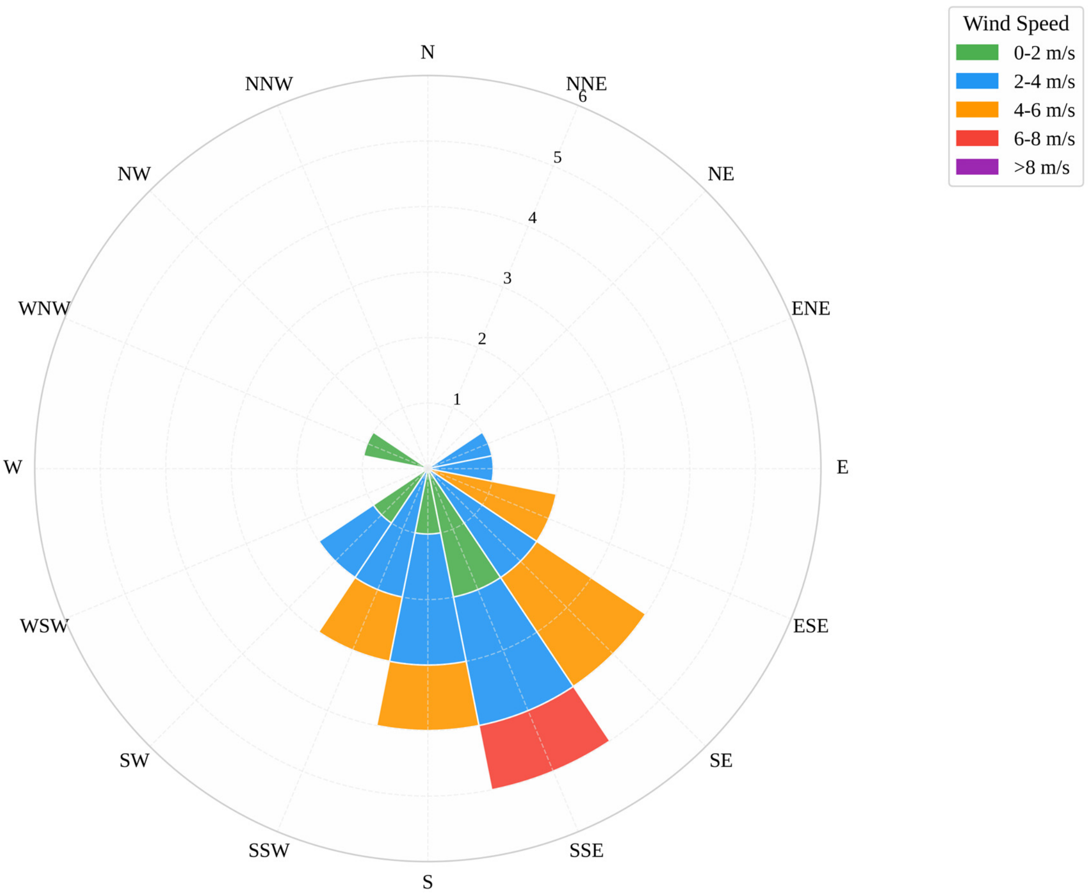

Figure S6. Wind rose diagram aggregated from all sampling stations in the Southern Yellow Sea during May 2021.

Note: Wind speed was categorized into five classes: [0, 2], (2, 4], (4, 6], (6, 8], and >8 m/s, with detailed information provided in Table 1. The data presented in this figure were obtained from in situ measurements of wind direction and wind speed at 23 stations in the Southern Yellow Sea during the cruise survey of the corresponding month. As the sampling period covered only selected days within the month, the limited spatial and temporal coverage means the results may not fully reflect the complete wind field characteristics for that month. It should be noted that the Southern Yellow Sea is extensive, and the pronounced thermal contrast between land and sea leads to significant diurnal variations in wind direction; therefore, the aggregated wind rose diagram cannot fully represent the local wind field characteristics at individual stations. For more detailed raw data, including daily mean wind speed, maximum wind speed, and corresponding wind direction at each station, users are advised to access the public database of the National Oceanic and Atmospheric Administration (NOAA) at https://www.ncei.noaa.gov/ to download observational records for the relevant period.

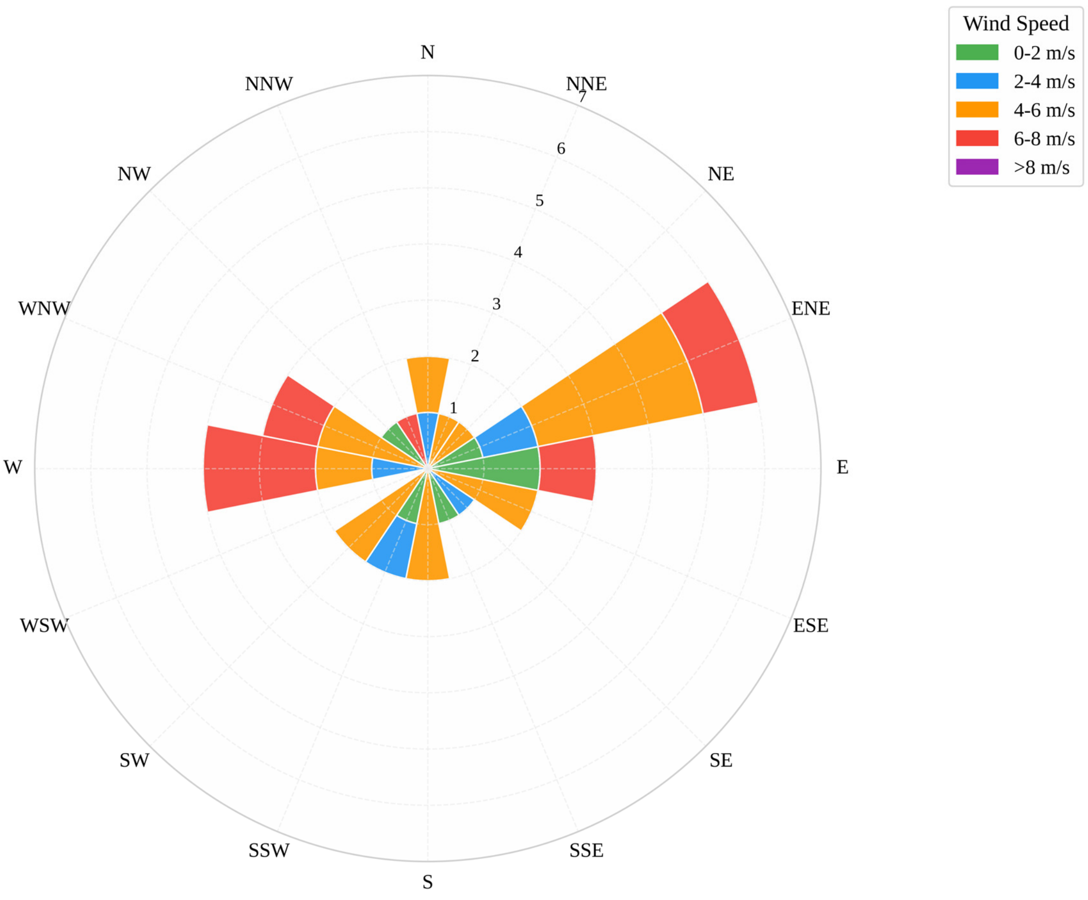

Figure S7. Wind rose diagram aggregated from all sampling stations in the Southern Yellow Sea during November 2021.

Note: Wind speed was categorized into five classes: [0, 2], (2, 4], (4, 6], (6, 8], and >8 m/s, with detailed information provided in Table 1. The data presented in this figure were obtained from in situ measurements of wind direction and wind speed at 32 stations in the Southern Yellow Sea during the cruise survey of the corresponding month. As the sampling period covered only selected days within the month, the limited spatial and temporal coverage means the results may not fully reflect the complete wind field characteristics for that month. It should be noted that the Southern Yellow Sea is extensive, and the pronounced thermal contrast between land and sea leads to significant diurnal variations in wind direction; therefore, the aggregated wind rose diagram cannot fully represent the local wind field characteristics at individual stations. For more detailed raw data, including daily mean wind speed, maximum wind speed, and corresponding wind direction at each station, users are advised to access the public database of the National Oceanic and Atmospheric Administration (NOAA) at https://www.ncei.noaa.gov/ to download observational records for the relevant period.

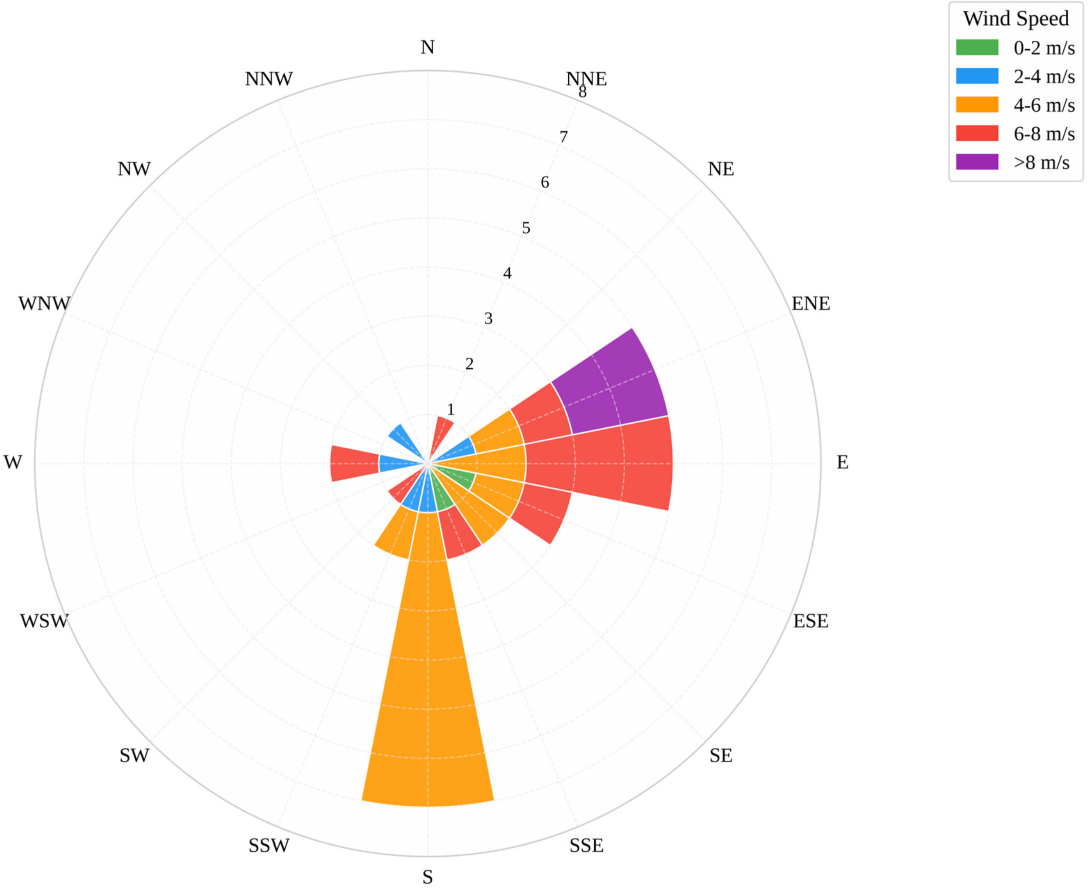

Figure S8. Wind rose diagram aggregated from all sampling stations in the Southern Yellow Sea during March 2022.

Note: Wind speed was categorized into five classes: [0, 2], (2, 4], (4, 6], (6, 8], and >8 m/s, with detailed information provided in Table 1. The data presented in this figure were obtained from in situ measurements of wind direction and wind speed at 31 stations in the Southern Yellow Sea during the cruise survey of the corresponding month. As the sampling period covered only selected days within the month, the limited spatial and temporal coverage means the results may not fully reflect the complete wind field characteristics for that month. It should be noted that the Southern Yellow Sea is extensive, and the pronounced thermal contrast between land and sea leads to significant diurnal variations in wind direction; therefore, the aggregated wind rose diagram cannot fully represent the local wind field characteristics at individual stations. For more detailed raw data, including daily mean wind speed, maximum wind speed, and corresponding wind direction at each station, users are advised to access the public database of the National Oceanic and Atmospheric Administration (NOAA) at https://www.ncei.noaa.gov/ to download observational records for the relevant period.

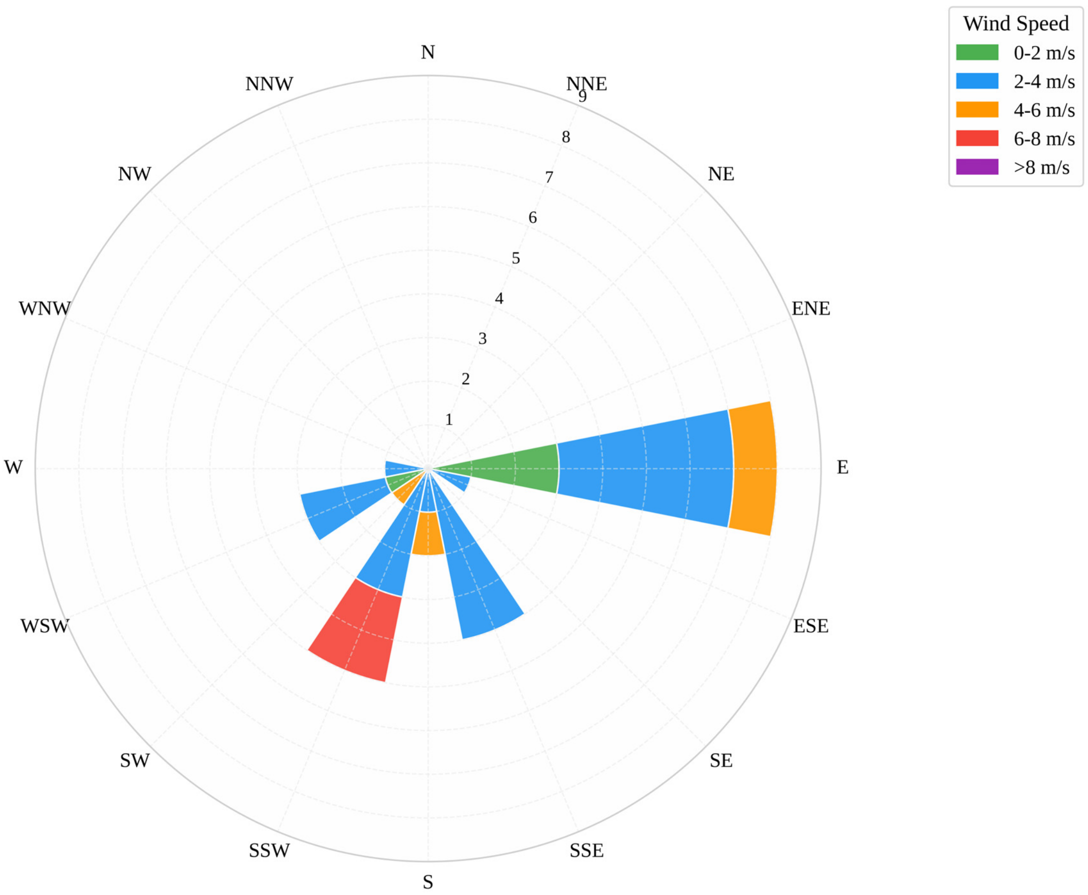

Figure S9. Wind rose diagram aggregated from all sampling stations in the Southern Yellow Sea during May 2022.

Note: Wind speed was categorized into five classes: [0, 2], (2, 4], (4, 6], (6, 8], and >8 m/s, with detailed information provided in Table 1. The data presented in this figure were obtained from in situ measurements of wind direction and wind speed at 25 stations in the Southern Yellow Sea during the cruise survey of the corresponding month. As the sampling period covered only selected days within the month, the limited spatial and temporal coverage means the results may not fully reflect the complete wind field characteristics for that month. It should be noted that the Southern Yellow Sea is extensive, and the pronounced thermal contrast between land and sea leads to significant diurnal variations in wind direction; therefore, the aggregated wind rose diagram cannot fully represent the local wind field characteristics at individual stations. For more detailed raw data, including daily mean wind speed, maximum wind speed, and corresponding wind direction at each station, users are advised to access the public database of the National Oceanic and Atmospheric Administration (NOAA) at https://www.ncei.noaa.gov/ to download observational records for the relevant period.

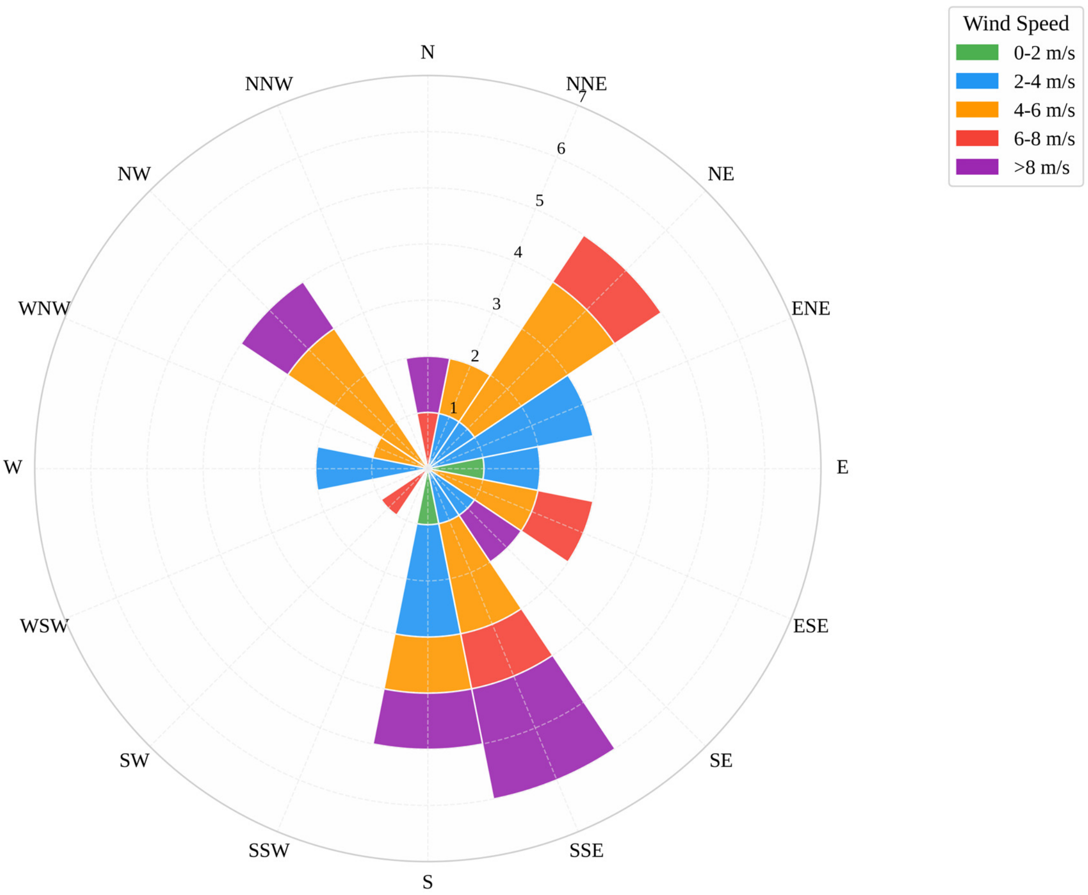

Figure S10. Wind rose diagram aggregated from all sampling stations in the Southern Yellow Sea during December 2022.

Note: Wind speed was categorized into five classes: [0, 2], (2, 4], (4, 6], (6, 8], and >8 m/s, with detailed information provided in Table 1. The data presented in this figure were obtained from in situ measurements of wind direction and wind speed at 38 stations in the Southern Yellow Sea during the cruise survey of the corresponding month. As the sampling period covered only selected days within the month, the limited spatial and temporal coverage means the results may not fully reflect the complete wind field characteristics for that month. It should be noted that the Southern Yellow Sea is extensive, and the pronounced thermal contrast between land and sea leads to significant diurnal variations in wind direction; therefore, the aggregated wind rose diagram cannot fully represent the local wind field characteristics at individual stations. For more detailed raw data, including daily mean wind speed, maximum wind speed, and corresponding wind direction at each station, users are advised to access the public database of the National Oceanic and Atmospheric Administration (NOAA) at https://www.ncei.noaa.gov/ to download observational records for the relevant period.
